# Single-cell RNA sequencing reveals ex vivo signatures of SARS-CoV-2-reactive T cells through ‘reverse phenotyping’

**DOI:** 10.1038/s41467-021-24730-4

**Published:** 2021-07-26

**Authors:** David S. Fischer, Meshal Ansari, Karolin I. Wagner, Sebastian Jarosch, Yiqi Huang, Christoph H. Mayr, Maximilian Strunz, Niklas J. Lang, Elvira D’Ippolito, Monika Hammel, Laura Mateyka, Simone Weber, Lisa S. Wolff, Klaus Witter, Isis E. Fernandez, Gabriela Leuschner, Katrin Milger, Marion Frankenberger, Lorenz Nowak, Katharina Heinig-Menhard, Ina Koch, Mircea G. Stoleriu, Anne Hilgendorff, Jürgen Behr, Andreas Pichlmair, Benjamin Schubert, Fabian J. Theis, Dirk H. Busch, Herbert B. Schiller, Kilian Schober

**Affiliations:** 1 Institute of Computational Biology, Helmholtz Zentrum München, Neuherberg, München, Germany; 2 TUM School of Life Sciences Weihenstephan, Technical University of Munich, Freising, Germany; 3 Institute of Lung Biology and Disease and Comprehensive Pneumology Center with the CPC-M bioArchive, Helmholtz Zentrum Muenchen, Member of the German Center for Lung Research (DZL), Munich, Germany; 4 Institute for Medical Microbiology, Immunology and Hygiene, Technische Universität München (TUM), Munich, Germany; 5 Institute of Virology, Technische Universität München (TUM), Munich, Germany; 6 Laboratory of Immunogenetics and Molecular Diagnostics, Department of Transfusion Medicine, Cell Therapeutic Agents and Hemostaseology, LMU Munich, Munich, Germany; 7 Department of Medicine V, University Hospital, LMU Munich, Comprehensive Pneumology Center Munich (CPC-M), Member of the German Center for lung research (DZL), Munich, Germany; 8 Center for Thoracic Surgery Munich, Ludwig-Maximilians-University of Munich (LMU) and Asklepios Lung Clinic Munich-Gauting, Munich and Gauting, Munich, Germany; 9 Asklepios Biobank for pulmonary diseases, Gauting, Germany; 10 Member of the German Center for Lung Research (DZL), Center for Comprehensive Developmental Care (CDeCLMU), Department of Neonatology, Perinatal Center, Munich, Germany; 11 German Center for Infection Research (DZIF), partner site Munich, Munich, Germany; 12 Department of Mathematics, Technical University of Munich, Garching, Germany; 13 Focus Group ‘Clinical Cell Processing and Purification”, Institute for Advanced Study, TUM, Munich, Germany; 14 Grosshadern, Hospital of the Ludwig-Maximilians University (LMU), Munich, Germany; 15 Present Address: Institute of Lung Biology and Disease, Comprehensive Pneumology Center, Helmholtz Zentrum München, Neuherberg, München, Germany; 16 Present Address: Microbiological Institute—Institute of Clinical Microbiology, Immunology and Hygiene, University Hospital of Erlangen, Erlangen, Germany

**Keywords:** Cellular immunity, Viral infection, T-cell receptor, SARS-CoV-2, Viral infection

## Abstract

The in vivo phenotypic profile of T cells reactive to severe acute respiratory syndrome (SARS)-CoV-2 antigens remains poorly understood. Conventional methods to detect antigen-reactive T cells require in vitro antigenic re-stimulation or highly individualized peptide-human leukocyte antigen (pHLA) multimers. Here, we use single-cell RNA sequencing to identify and profile SARS-CoV-2-reactive T cells from Coronavirus Disease 2019 (COVID-19) patients. To do so, we induce transcriptional shifts by antigenic stimulation in vitro and take advantage of natural T cell receptor (TCR) sequences of clonally expanded T cells as barcodes for ‘reverse phenotyping’. This allows identification of SARS-CoV-2-reactive TCRs and reveals phenotypic effects introduced by antigen-specific stimulation. We characterize transcriptional signatures of currently and previously activated SARS-CoV-2-reactive T cells, and show correspondence with phenotypes of T cells from the respiratory tract of patients with severe disease in the presence or absence of virus in independent cohorts. Reverse phenotyping is a powerful tool to provide an integrated insight into cellular states of SARS-CoV-2-reactive T cells across tissues and activation states.

## Abstract (web summary)

High resolution characterisation of the virus specific T cell response to SARS CoV2 provides further understanding to the immune response to the infection. Here the authors apply a reverse phenotyping approach to interrogate the SARS-CoV-2 T cell compartment at single cell resolution.”

## Introduction

COVID-19 is a new form of viral pneumonia^1^ caused by SARS-CoV-2^2^ and is affecting 67,073,749 patients, with 1,536,072 deaths, worldwide (source: Johns Hopkins University, as of December 7, 2020). Adaptive immunity is swiftly induced upon COVID-19 infection^3^. T cells play a central role in this process and are implicated in contributing both to long-lasting immunity as well as to putative immunopathology^4,5^. A thorough understanding of T cell responses to SARS-CoV-2 is therefore urgently needed.

Immunodominant SARS-CoV-2 antigen specificities have been identified with unprecedented speed for an emerging pathogen, and phenotypic characterization of antigen-reactive T cells has quickly been performed by a plethora of studies^6–16^. While there is general agreement that SARS-CoV-2-reactive T cells are activated and differentiate during the course of the immune response, the extent of activation and differentiation are controversial^4,5^.

Vast resources of deeply profiled immune cells from COVID-19 patients do already exist and are bundled e.g., by the Human Cell Atlas initiative (www.humancellatlas.org). It is difficult, however, for any single study to cover different patient disease states and sample sites, together with detailed clinical metadata and deep profiling down to single-cell resolution, including antigen-specific analyses. For example, few studies have investigated T cell states across different tissue sites (e.g., from peripheral blood (PB) and the respiratory tract) in identical COVID-19 patients^5^. Generally, antigen-specific T cells are expected to have different activation states in different tissues depending on the course of the disease. When a viral antigen is still present in the respiratory tract, T cells at the site of infection should show a more activated phenotype compared to PB T cells which are not being activated by the antigen at the time of analysis. In this context, difficulties to clearly define the phenotypic profile of currently and previously activated SARS-CoV-2-reactive T cells also hinder the precise contextualization of T cell signatures across publicly available data sets.

Methodologies for the characterization of antigen-reactive T cells differ and thereby obscure a clear insight into T cell phenotypes. Some reports have described phenotypes of activated T cells during the course of COVID-19 without assessment of *bona fide* antigen specificity, whereas other studies restricted phenotypic characterization to antigen-reactive T cells^4^. Although such restriction to antigen-reactive T cells is clearly desired, it entails methodological challenges on its own: initial detection of antigen-reactive T cells usually requires in vitro re-stimulation with SARS-CoV-2 antigens. Upon re-stimulation, antigen-reactive cells can be defined through upregulation of activation markers (such as CD154) or release of effector cytokines (such as IFNγ)^17^. This, however, automatically also introduces major phenotypic biases for any downstream profiling since in vitro activated T cells show different phenotypes compared to unperturbed cells (i.e., cells as they are in vivo; technically, any analysis outside the body is ex vivo; we therefore here refer to unstimulated cells from our experiments as “ex vivo”; in contrast, we use the term “in vitro”, when cells are additionally re-stimulated since the stimulation step introduces major differences compared to the in vivo setting; finally, we use the term “in vivo” when the actual in vivo setting is referred to)^18^. In order to address these well-known challenges, peptide human leukocyte antigen (pHLA) multimers have been developed^19^. Through removal of specifically designed pHLA multimers (“Streptamers”), unperturbed cellular phenotypes of antigen-reactive T cells can be even completely preserved^20^. Yet, pHLA multimer technology requires a previous definition of specific epitopes and is restricted to individual HLA haplotypes. Furthermore, pHLA multimers are often difficult to generate for HLA class II-restricted CD4 T cells^21^. These limitations represent a significant technical obstacle to investigating unbiased in vivo phenotypic profiles of antigen-reactive T cells.

Single-cell RNA sequencing (scRNA seq) allows simultaneous analysis of the global cellular transcriptome as well as identification of T cell receptor (TCR) sequences^22,23^. Recently, it has been demonstrated that scRNA seq can be used to reveal activation-induced phenotypic profiles of antigen-reactive T cells^24^. Samples can be split up after isolation from the tissue, stimulated in vitro in an antigen-specific manner (and, for comparison, left unstimulated), and then sequenced. The natural TCR can thereby serve as a barcode to link T cells of the activated and unstimulated condition belonging to the same in vivo expanded clonotype with a common antigen specificity.

Here, we show that such “reverse phenotyping” (Supplementary Fig. 1a) can be used to identify and characterize SARS-CoV-2-reactive T cells from PB of severely diseased COVID-19 patients. Furthermore, an integrated analysis of respiratory material from our own and independent reference cohorts shows that transcriptomic states of respiratory T cells from virus-positive patients are most similar to in vitro stimulated reactive T cells from PB, consistent with a “hot” phenotype driven by acute activation at the site of infection. Thus, we present transcriptional shifts from acute disease to resolution after virus clearance in antigen reactive CD4 and CD8 T cells during the course of COVID-19.

## Results

### Patient characteristics and experimental set-up for “reverse phenotyping”

**Fig. 1:**
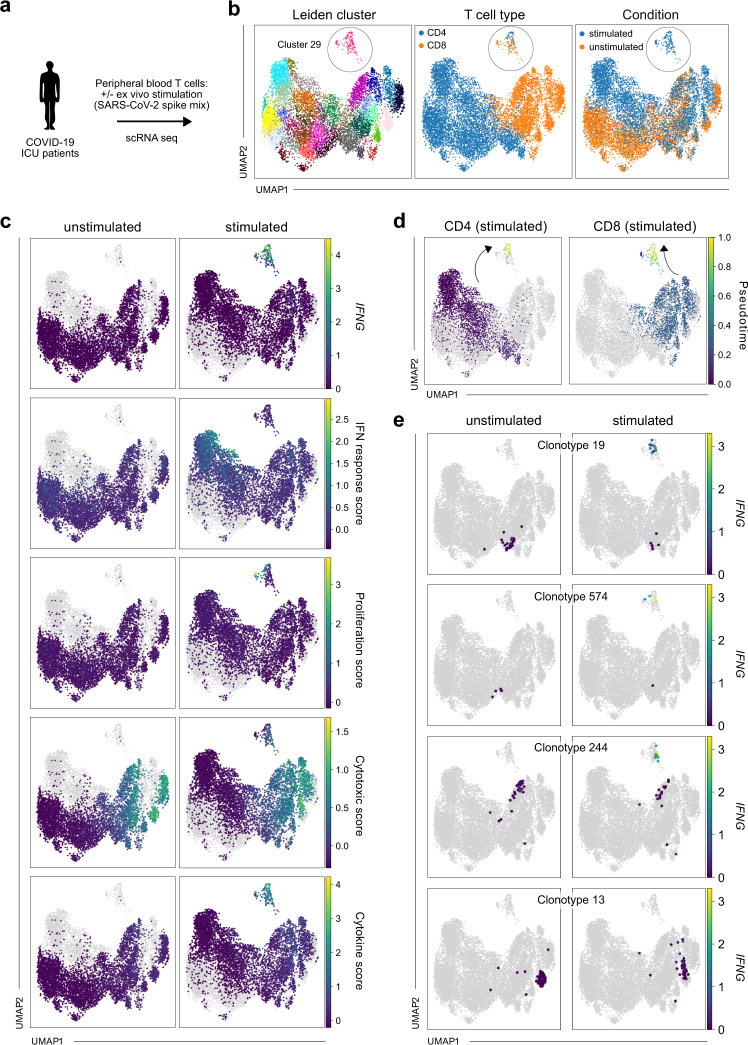
Single-cell RNA sequencing allows detection of transcriptional shifts induced by antigen-specific stimulation in TCR-barcoded clonotypes. **a** Experimental setup; cells from tracheal aspirates and peripheral blood T cells (flow cytometry-sorted for CD4 or CD8 positivity) of intensive care unit (ICU) patients with COVID-19 were profiled by single-cell RNA sequencing (scRNA seq). Before scRNA seq, peripheral blood T cells were stimulated with SARS-CoV-2 spike protein–peptide mix or left untreated. **b** Uniform manifold approximation and projection (UMAP) of Leiden clusters (left panel), CD4 and CD8 T cells (middle panel), and stimulated and unstimulated T cells (right panel) (*n* = 11,460 cells in total). **c** *IFNG* expression, IFN response score, proliferation score, cytotoxic score, and cytokine score (from top to bottom) in unstimulated (left panels; stimulated cells shown in gray) or stimulated (right panels; unstimulated cells shown in gray) T cells (*n* = 11,460 cells in total). **d** Stimulated CD4 and CD8 T cells with highlighted pseudotime after defining the endpoint of pseudotime at the tip of cluster 29 (*n* = 11,460 cells in total). **e** *IFNG* expression in unstimulated or stimulated T cells for four representative clonotypes. For each clonotype, cells belonging to that clonotype are shown in an individual panel pair (cells from the unstimulated condition in left panels, cells from the stimulated condition in right panels), while cells not belonging to that clonotype are shown in gray (*n* = 11,460 cells in total, 43 cells for CD4 clonotype 19, 8 for CD4 clonotype 574, 61 for CD8 clonotype 244, 165 for CD8 clonotype 13). Data are shown for patient GT_3.

We acquired respiratory material (TAs) as well as PB mononuclear cells (PBMCs) from severely diseased patients with COVID-19 (Fig. 1a; Supplementary Data 1). Consistent with the risk profile for severe disease, seven of nine patients in this “Munich cohort” were male and age ranged from 51 to 82 years (median 79 years). All patients were treated in an intensive care unit (ICU) and had been on a respirator for 8–38 days (median 29 days) at the time of sampling. After initial detection of SARS-CoV-2 via polymerase chain reaction (PCR), eight of nine patients had turned consistently virus-negative or had at least one prior negative test result at the time of sampling. Ultimately, two patients deceased and seven patients recovered. ScRNA seq (3′ transcriptomics) was performed on TAs from all nine patients. For two patients (“GT_3” and “GT_2”), we stimulated PBMCs with SARS-CoV-2 spike protein–peptide mix or left control samples unstimulated, and likewise performed scRNA seq (5′ transcriptomics and VDJ) after flow cytometry-assisted cell sorting of >10.000 CD4 and CD8 T cells for each patient.

### Antigen-induced transcriptional shifts in PBMCs

After preprocessing, uniform manifold approximation and projection (UMAP)^25^ of stimulated and unstimulated T cells identified a group of cells in both patients which were separate from all other cells (Leiden cluster 29 in patient GT_3; Fig. 1b). The cluster encompassed both CD4 and CD8 cells and consisted almost exclusively of cells from the stimulated condition. We hypothesized that this distinct stimulation-induced cluster represented antigen-reactive T cells. Indeed, only the cells from that cluster upregulated *IFNG* (the gene encoding Interferon-γ, IFNγ) after stimulation (Fig. 1c). Antigen-reactive CD4 Th cell lineages (particularly those that are not Th1 cells) do not necessarily express IFNγ in response to antigen^21^. For this reason, and in order to not focus on upregulation of a single gene, we explored more holistic and unbiased antigen-reactive response scores for CD4 and CD8 T cells, as previously identified by scRNA seq after aCD3/aCD28 stimulation^18^. Upon stimulation, CD4 T cells have been described to show sequentially enriched IFN response (early activation) and proliferation (late activation) scores^18^. Accordingly, in our analysis CD4 T cells showing high proliferation scores were exclusively present upon stimulation, and only in cluster 29 (Fig. 1c). Interestingly, particularly CD4 cells outside the cluster (on the left side of the UMAP) underwent a general transcriptional shift through stimulation, whereas this did not occur for CD8 cells. These seemingly unspecifically shifting CD4 T cells were also the ones that showed a high IFN response (early activation) score (Fig. 1c) or, e.g., expression of the IFNγ receptor gene *IFNGR2* (Supplementary Fig. 1b) upon stimulation. This confirms previous observations of differential CD4 transcriptomics through sensing of TCR-triggered cytokines such as IFNγ^18^. The source of the sensed cytokines could be antigen-reactive CD4 or CD8 T cells that are neighboring and stimulated in the culture system.

CD8 T cells have been described to undergo sequential transcriptional states upon activation that are reflected by high “cytotoxic” (early activation) or “cytokine secretion” scores (late activation)^18^. While in our analysis almost all CD8 T cells showed a high cytotoxic score, high cytokine scores were exclusively confined to cells belonging to the antigen-reactive cluster 29 in the stimulated condition (Fig. 1c). Differential gene expression analysis confirmed that cluster 29 CD8 T cells showed upregulation of genes like *IFNG*, *TNF*, *IL2*, *CCL3*, *CCL4*, or *GZMB* (Supplementary Data 2 and 3), in line with CD8 T cell activation. We next aimed to elucidate if activation-induced transcriptomic changes followed consistent gradients. To this end, we defined the tip of cluster 29 as an “endpoint” in pseudo-time and ordered all stimulated cells along this trajectory of transcriptomic similarity (Fig. 1d). This shows that our approach could resolve a spectrum of activation states across clonotypes, feeding into cluster 29 from different directions. We validated our findings in the second severely ill patient (“GT_2”) with COVID-19 for which we stimulated or did not stimulate PB CD4 and CD8 T cells with SARS-CoV-2 spike protein–peptide mix. Again, antigen stimulation-induced transcriptional shifts giving rise to a specific “reactive Leiden cluster” (in this patient cluster 36; Supplementary Fig. 2a, b). Data integration showed that the reactive Leiden clusters from both patients showed convergent phenotypes (Supplementary Fig. 2c). The reactive clusters constituted 0.39% (cluster 36 in patient GT_2) or 2.88% (cluster 29 in patient GT_3) of all stimulated cells and were specific to the stimulated condition (Supplementary Fig. 2d).

### Identification of antigen-reactive clonotypes

Using the TCR information from VDJ sequencing, we next investigated whether differential transcriptional responses to antigen stimulation could be attributed to specific clonotypes. We detected CDR3αβ sequences in 92% of analyzed T cells (69.9% fully paired CDR3αβ; 3.4% CDR3α only; 26.7% CDR3β only). In order to ensure the clonotypic nature of the investigated cells, we first only included clonotypes based on identical fully paired CDR3αβ sequences in our analysis. For both CD4 and CD8 T cell clonotypes, we recovered similar cell numbers in the stimulated and non-stimulated conditions (Supplementary Fig. 3a, c). This enabled us to identify clonotypes that underwent transcriptional shifts upon antigenic stimulation (Fig. 1e). Interestingly, we observed a heterogeneous response pattern within clonotypes, with some cells moving into the antigen-reactive cluster 29 (with concomitant upregulation of *IFNG*) and other cells staying in a transcriptional state that is consistent with their ex vivo phenotype without in vitro re-stimulation (Fig. 1e; Supplementary Fig. 3e). Possibly, these response patterns are a result of stochastic antigen encounters in the experimental stimulation system in vitro. The remarkable degree of transcriptional synchronization specific for each TCR ex vivo confirms previous observations of TCR-mediated phenotypic imprinting^26,27^ and renders high credibility to the assessment of clonotypes that are as small as a few cells per condition group (such as clonotype 574; Fig. 1e). Overall, the distinct transcriptional shifts induced by antigenic stimulation in specific clonotypes suggested the presence of antigen-reactive T cells.

**Fig. 2:**
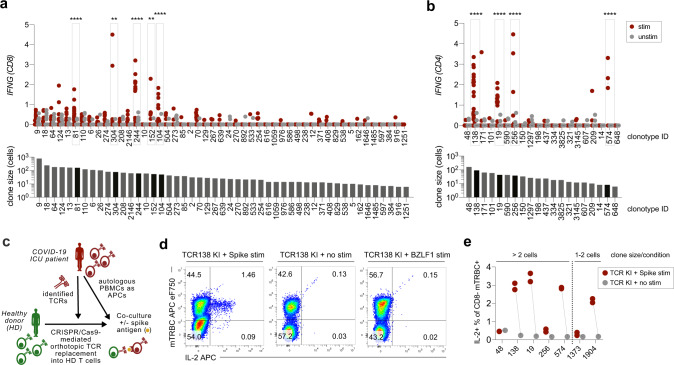
Identification and validation of SARS-CoV-2 antigen-reactive T cell receptors. **a** Top: *IFNG* expression of CD8 clonotypes after antigenic or no stimulation. Only clonotypes with at least three cells in each condition and as defined by a unique αβ CDR3 sequence were included in this analysis. Each dot represents one cell and lines indicate the median. *n* = 56/66 (clone 81), 38/30 (clone 304), 25/30 (clone 244), 16/16 (clone 152), and 23/22 (clone 104) cells for stim/unstim condition, respectively. Exact cell numbers per clonotype can be found in Supplementary Data 11. Antigen-reactive clonotypes (defined through statistically significant upregulation of *IFNG*) are highlighted. *p* < 0.0001 for clones 81, 244, and 104; *p* = 0.0033 for clone 304. Bottom: Clonotype sizes in numbers of total cells analyzed. Clonotypes defined as “reactive” are highlighted in black. **b** As in a, but for CD4 clonotypes. *n* = 28/42 (clone 138), 17/26 (clone 19), 11/18 (clone 256), and 4/4 (clone 574) cells for stim/unstim condition, respectively. *p* < 0.0001 for clones 138, 19, 256, and 574 **c** Experimental setup; T cells from healthy donors were equipped with TCRs identified from COVID-19 patients by CRISPR/Cas9-mediated orthotopic TCR replacement (OTR); transgenic T cells were co-incubated with antigen-loaded patient PBMCs and reactivity was investigated by intracellular cytokine staining. **d** Flow-cytometric analysis of antigen-stimulated TCR-engineered T cells, 1 week after OTR; representative data are shown for CD4 TCR138 after stimulation with SARS-CoV-2 spike protein–peptide mix, no stimulation, and stimulation with irrelevant EBV antigen BZLF1 peptide mix (negative controls); mTRBC: murine constant region of the TCR beta chain incorporated into transgenic TCRs for detection; shown gates are pre-gated for CD3+ CD8− living lymphocytes. **e** Quantification of spike antigen-specific reactivity for selected clonotypes tested in (**b**) as well as two additional small clonotypes; for antigen-specific transcriptional shifts detected by initial scRNA seq for the respective clones see Fig. 1e, Supplementary Fig. 5a and Supplementary Fig. 7b. *n* = 1 technical replicate (no stim) and *n* = 2 technical replicates (stim). Statistical analysis by two-way ANOVA (**** each for the treatment effect and clonotype distribution) followed by Sidak’s multiple comparisons test *p < 0.05, **p < 0.01, ***p < 0.001, ****p < 0.0001 (**a**, **b**). Data are shown for patient GT_3.

While mean proliferation scores (for CD4 T cells) or mean cytokine scores (for CD8 T cells) generally correlated with mean *IFNG* expression per clonotype after antigen-specific stimulation (Supplementary Fig. 3b, d), *IFNG* expression seemed to be the more sensitive read-out. We, therefore, decided to define antigen-reactive T cells by significantly enhanced *IFNG* expression after antigen-specific stimulation (Fig. 2a, b). Among clonotypes with at least three cells in each stimulation condition, we detected five and four antigen-reactive clonotypes for CD8 and CD4 T cells, respectively. Apart from clonotype 574, reactive clonotypes were large in size, consistent with previous in vivo activation and clonal expansion.

This first analysis was strictly restricted to paired αβ TCRs. We also explored all clonotypes defined by unique double (CDR3αβ) *or* single (CDR3α or CDR3β) chain sequences (Supplementary Fig. 4). This yielded two further CD8 clonotypes (clonotypes 651 and 548). Upon further examination, these single-chain (CDR3β only) clonotypes shared CDR3β sequences with clonotypes 81 and 244, respectively, and showed similar *IFNG* upregulation (Supplementary Fig. 4a) and global transcriptomic states ex vivo and upon stimulation (Supplementary Fig. 4b) compared to their paired partner clonotypes. We also identified an additional single-chain CD4 clonotype (clonotype 136), for which no paired partner clonotype existed (Supplementary Fig. 4c). These findings are best explained by the technical variability of CDR3α sequence detection. Since we conversely did not find any false-positive pairing of TCRs sharing the same CDR3β, but different CDR3α sequences, we included these single-chain clonotypes into downstream phenotypic analyses.

Having defined reactive clonotypes through statistically significant upregulation of *IFNG* after stimulation (based on a two-way ANOVA followed by Sidak’s multiple comparisons test), we aimed to ensure we would not miss clonotypes that show antigen-dependent reactivity through other means than *IFNG* upregulation. We therefore again analyzed cytokine/cytotoxicity scores for CD8 T cells and proliferation/IFN response scores for CD4 T cells in a clonotype-dependent manner. While for CD8 T cells, no clonotype-dependent enrichment was visible after stimulation in the cytotoxicity score (early activation), in the cytokine scores (late activation) we detected broad statistically significant signals, which did not mirror global transcriptomic shifts in sanity checks and therefore seemed to reflect rather unspecific changes (e.g., compare statistically significant results for clonotype 13 in Supplementary Fig. 4a with missing reactivity in Fig. 1e). In CD4 T cells, clonotype and stimulation-dependent IFN responses (early activation) likewise yielded inconsistent results (Supplementary Fig. 4c), whereas the proliferation score (late activation) identified the same antigen-reactive clonotypes as defined through *IFNG* upregulation except for one clonotype, which was missed by the proliferation score (Supplementary Fig. 4c). Finally, targeted exploration of markers alternative to *IFNG*, such as *MKI67*, *TNFRSF9* (coding for CD137), IL-2, IL-4, IL-5, IL-9, IL-10, IL-13, IL-17, or IL-22 did not yield any statistically significant antigen-induced upregulation in a clonotype-dependent manner.

To capture the global antigen-specific reactivity landscape of the TCR repertoire, we calculated correlations between clonotypes in terms of the general transcriptomic shift these clonotypes underwent upon stimulation. This revealed two groups of clonotypes that showed high inter-clonotype correlations within the respective group (Supplementary Fig. 5a). The larger group consisted mostly of CD8 clonotypes assessed as non-reactive to antigen based on the lack of statistically significant *IFNG* upregulation and recruitment into cluster 29. The few clonotypes that are antigen-reactive and/or belong to cluster 29 in that larger group may represent weakly cross-reactive clonotypes. A smaller group contained almost exclusively CD4 and CD8 clonotypes that we defined as antigen-reactive based on statistically significant *IFNG* upregulation (Supplementary Fig. 4) and/or were present in the stimulation-induced cluster 29 (Fig. 1b).

In order to experimentally validate the antigen reactivity of our selected clonotypes, we generated TCR-transgenic T cells by CRISPR/Cas9-mediated orthotopic TCR replacement (OTR)^28^. Through this technology, transgenic TCRs are knocked into the endogenous TCR gene locus, thereby simultaneously placing the transgenic TCR under physiological transcriptional control and knocking out the endogenous TCR. We equipped healthy donor T cells by OTR with identified CD4 TCRs (Fig. 2c). From our screening of clonotypes with at least three cells in each condition (Fig. 2b), we included the four TCRs defined as reactive (TCRs 138, 19, 256, and 574) and the TCR from the largest CD4 clonotype (TCR 48), which did not show antigen-induced transcriptomic changes. After TCR knock-in (KI), T cells with TCRs that were previously defined as reactive (TCRs 138, 19, 256, and 574) all showed SARS-CoV-2 spike antigen-dependent reactivity, whereas TCR 48 KI T cells did not (Fig. 2d, e). To test the sensitivity of our system, we additionally investigated TCRs from two very small clonotypes (1373 and 1904), which were not included in the initial definition of reactive clonotypes (Fig. 2b) since they had less than three cells in each condition. Remarkably, we could validate reactivity for TCR 1904, which had a transcriptional shift into cluster 29 with concomitant *IFNG* upregulation in one of two cells in the stimulated condition, whereas reactivity for TCR 1373 was missing, as predicted based on the lack of *IFNG* upregulation and no movement into cluster 29 (Fig. 2e, Supplementary Fig. 5b). Overall, these data reinforced our approach to using stimulation-induced *IFNG* upregulation as a surrogate marker to detect antigen-reactive clonotypes and functionally validated SARS-CoV-2 reactive TCRs.

### Reverse phenotyping reveals in vitro antigen re-stimulation-induced phenotypic biases in PB T cells

The identification of antigen-reactive clonotypes enabled us to perform “reverse phenotyping” by “looking back” at the phenotype without re-stimulation for clonotypes that undergo defined functional changes after re-stimulation, using their TCR sequence as natural barcodes. In other words, this enabled us to define antigen-reactive clonotypes by a functional read-out, and then investigate the phenotype of these cells *would have had* if they had not been stimulated. Through this, we can on the one hand investigate systematic phenotypic effects that are introduced by antigenic re-stimulation in vitro, and on the other hand, explore the unperturbed ex vivo phenotype of antigen-reactive T cells.

**Fig. 3:**
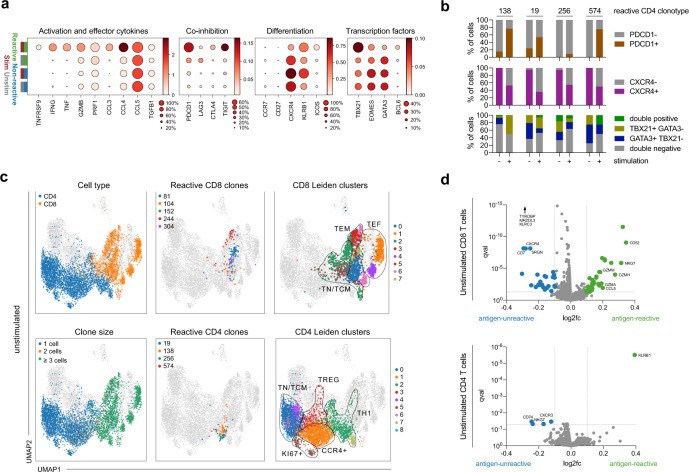
Reverse phenotyping reveals systematic biases induced by antigenic stimulation and allows precise definition of ex vivo transcriptional profiles of SARS-CoV-2 antigen-reactive T cells. **a** Dot plots of log-normalized expression of selected marker genes by clonotype group (reactive and non-reactive) and by condition (stimulated: “stim”, unstimulated: “unstim”) for CD4 T cells (stimulated reactive *n* = 68, unstimulated reactive *n* = 178, unstimulated nonreactive *n* = 539, stimulated nonreactive *n* = 249). **b** Fraction of gene-positive cells with >0 UMIs on the presented gene by clonotype and condition (+: stimulated, −: unstimulated). **c** UMAPs indicating cells from the unstimulated condition in colors with cells from the stimulated condition shown in gray (*n* = 11,460 cells in total); CD4 or CD8 T cell state (top left panel), clonotype size as defined through cell number (bottom left panel); reactive CD8 clonotypes as defined in (**a**) (top middle panel) or reactive CD4 clonotypes as defined in (**b**) (bottom middle panel), with all other cells not belonging to that clonotype also shown in gray; CD8 (top right panel) and CD4 (bottom right panel) Leiden clusters, including manual annotation of cluster groups for the sake of clarity. **d** Volcano plots of differential expression test of cells from non-reactive versus reactive clonotypes in unstimulated CD8 (top) and CD4 (bottom) T cells. For the sake of clarity, *TYROBP* (l2fc −0.48; qval 7 × 10^−131^), *KIR2DL3* (l2fc −0.15; qval 3 × 10^−60^), and *KLRC3* (l2fc −0.13; qval 3 × 10^−^^55^) are not displayed (CD8). Data are shown for patient GT_3.

Upon re-stimulation in vitro, antigen-reactive CD4 T cells upregulated *TNFRSF9* (encoding CD137) and effector cytokines: *IFNG*, *TNF*, *XCL1*, *XCL2*, or *CCL3* were absent in unstimulated reactive CD4 T cells, but strongly induced upon stimulation (Fig. 3a, Supplementary Fig. 6a). *GZMB* or *CCL4* were expressed in reactive CD4 T cells (as well as in non-reactive cells) in the unstimulated condition already, but the expression was boosted by stimulation for reactive CD4 T cells only (Fig. 3a). Of note, co-inhibitory molecules, such as PD-1 (*PDCD1*), *LAG3*, or *TIGIT*, were almost absent on antigen-reactive cells before stimulation, and only induced thereafter, indicating that detection of these molecules in in vitro stimulated T cells reflects T cell activation rather than exhaustion.

Indeed, many induced molecules are known to be differentially regulated after antigenic stimulation but also serve as important determinants of more stably committed T cell phenotypes. High expression of *ICOS*, *PDCD1*, and *TIGIT* has previously been described as a hallmark of SARS-CoV-2 specific CD4 T cells during severe disease^4^, but our findings indicate that the degree of expression can be significantly overestimated due to confounding stimulation effects. Conversely, expression of *CXCR4* or *KLRB1* would have been underestimated if cells had only been assessed after stimulation. CXCR4 is known to be more expressed in less differentiated T cells^29^, but downregulated upon T cell activation^30^. We next wondered whether and how in vitro stimulation with antigen also changed Th lineage-defining transcription factors, since these are particularly relevant for the assignment of CD4 T cell identity. Despite their robust *IFNG* upregulation upon antigenic stimulation, reactive CD4 T cells showed overall less Th1-defining *TBX21* (T-bet) expression ex vivo than it would have seemed after stimulation (Fig. 3a). Vice versa, expression of *EOMES* and Th2-defining *GATA3* was downregulated in reactive CD4 cells after antigenic stimulation (Fig. 3a). Antigen stimulation also induced expression of (circulating) follicular helper T cell ((c)Tfh)-defining *BCL6* in a “false-positive” manner in a few antigen-reactive CD4. Overall, we revealed that stimulation-induced broad changes in transcriptional profiles, ranging from expected upregulation of activation markers to more unexpected and complex changes in markers associated with stable phenotypes.

The mentioned observations account for comparisons of stimulated vs. unstimulated and reactive vs. non-reactive clonotypes in bulk (Fig. 3a). However, individual clonotypes have also different phenotypes a priori, and could therefore show unique reactivity upon stimulation. To explore this further, we analyzed stimulation-induced transcriptional changes for individual reactive clonotypes. This revealed expected as well as unexpected, consistent as well as heterogeneous phenotypic changes for reactive clonotypes. For example, while all reactive clonotypes upregulated *PDCD1* or downregulated *CXCR4* upon stimulation in a synchronized manner, stimulation-induced clonotype-specific changes in CD4 lineage defining *TBX21* and *GATA3* (Fig. 3b). Clonotype 138 upregulated and clonotype 256 downregulated *TBX21* expression after stimulation. In addition to this inter-clonal heterogeneity, intra-clonal variability adds an additional layer of complexity (Fig. 3b) since individual clonotypes can show mixed phenotypes^31^. Indeed, all reactive CD4 clonotypes contain cells that express *TBX21* and/or *GATA3*. We could however not detect a statistical dependency between *TBX21* and *GATA3* expression (see “Methods” section), suggesting that their expression is independent or that correlation could not be resolved.

Reactive CD8 T cells were overall less affected by antigenic stimulation than reactive CD4 T cells. After stimulation, CD8 T cells upregulated *IFNG* or *CCL4*, and also appeared more *LAG3* or *PDCD1* expressing than they were ex vivo (Supplementary Fig. 6b). In previous studies, these latter molecules have been described to be strongly increased in SARS-CoV-2 specific CD8 T cells during severe disease^4^, providing further evidence that phenotypes of T cells after in vitro re-stimulation with antigen should be interpreted with caution.

To further characterize the stimulation-induced changes of antigen-reactive CD4 and CD8 clonotypes, we performed differential expression analysis (Supplementary Fig. 6c, d; Supplementary Data 4 and 5). In CD4 T cells, this confirmed stimulation-induced upregulation of genes associated with induction of T cell activation^32–35^, or downregulation of genes known to be associated with repression of T cell activation^35–37^, but also revealed more unexpected differential gene expression of the Treg marker *FTH1*^38,39^, or of *TMSB4X* which is associated with effector-like Th1 cells^40,41^. In CD8 T cells, stimulation induced *GZMB* or *PRF1* most strongly, but was also associated with a prominent signature of actin (-binding) genes, which has previously described effector rather than exhausted T cells^32^. Of note, no distinct group of cells with a high tissue residency score was apparent in PB (Supplementary Fig. 6e).

In summary, reverse phenotyping unraveled expected and unexpected phenotypic effects introduced by antigenic re-stimulation in vitro. The results thereby also highlight transcriptional differences between currently and previously activated T cells. Beneath the surface of global transcriptomic shifts on the population level, single-cell resolution together with TCR barcoding demonstrates additional inter- and intra-clonal heterogeneity.

### Unperturbed ex vivo phenotypes of SARS-CoV-2-reactive T cells in PB

After comparing the differences between stimulated and unstimulated reactive clonotypes, we next focused on the unperturbed ex vivo phenotypes of SARS-CoV-2-reactive T cells in further detail by comparing them with unreactive clonotypes in the unstimulated condition only. Reactive CD4 T cells selectively stemmed from a T cell effector memory (TEM) Th1-like group of cells, which was transcriptionally similar to CD8 T cells (Fig. 3c, Supplementary Fig. 7a–d). Two CD4 clusters (CD4 clusters 2 and 7) entailed Th1 cells. Both clusters were the only ones harboring expanded clonotypes (Supplementary Fig. 7e), were high in *IL7R* and *CXCR4*, but low in *CCR7* and *CD27*, and were the only clusters that expressed *CX3CR1*, speaking for a TEM-like phenotype. (Supplementary Fig. 7f–h). Remarkably, reactive CD4 clonotypes were exclusively found in CD4 cluster 2, but not in cluster 7. Cluster 7 consisted of cells belonging to a single clonotype (number 48, the largest clonotype found for CD4 T cells; Fig. 2b). The similar phenotype, but lacking reactivity of this clonotype could indicate that clonotype 48 recognizes an entirely different target or a different part of SARS-CoV-2 (non-spike).

Reactive CD8 T cells were more evenly distributed across different CD8 TEM-like Leiden clusters (Fig. 3c, Supplementary Fig. 7). Of note, most CD8 T cells and the CD4 Th1 clusters showed clonal expansion, whereas few CD8 T cells and all non-Th1 CD4 clusters were clonotypically highly diverse (Fig. 3c), consistent with their overall little differentiated IL7R+ CCR7+ phenotype (Supplementary Fig. 7f).

To precisely define the ex vivo phenotypes of SARS-CoV-2-reactive T cells in an unbiased manner, we performed differential gene expression analysis comparing reactive and non-reactive clonotypes in the unstimulated condition (Supplementary Data 6 and 7). This identified *KLRB1* to be most significantly upregulated in reactive CD4 T cells (Fig. 3d). *KLRB1* encodes CD161 and is part of cytotoxic/Th 1 anti-viral T cells^42^. It has also already been described to be upregulated in SARS-CoV-2 reactive T cells after antigenic re-stimulation in vitro^43^. Together with IL7R expression, *KLRB1* also marks MAIT cells, but the TRAV/TRAJ expression of our antigen-reactive TCRs did not reflect invariant chains expressed by MAIT cells^27^.

Antigen-reactive CD8 T cells were characterized by high expression of granzymes, *CCL5* or the cytotoxic marker *NKG7*^18^, as well as *CD52*, the target of Alemtuzumab, for which a paracrine suppressive function is described in activated CD4 T cells^44^ (Fig. 3d). Antigen-reactive CD8 T cells also were markedly lower in *TYROBP*, *KIR2DL3*, and *KLRC3*. *TYROBP* encodes DAP12 and has known activating as well as inhibitory immune cell signaling roles when pairing with receptors belonging to the killer inhibitory receptors (KIR) or killer lectin-like receptor (KLR) family^45^. In CD8 T cells, TYROBP and KLR transcripts have previously been shown to be downregulated after activation^46^.

Using these differentially expressed genes of reactive and non-reactive cells in the unstimulated condition, we defined transcriptional signature scores characteristic for antigen-reactive CD4 or CD8 T cells ex vivo. These “ex vivo signature scores” overlapped to some extent with “stimulation-biased signature scores” that we defined by using differentially expressed genes of reactive and non-reactive cells in the stimulated condition (Supplementary Data 8 and 9), but also showed differences (Supplementary Fig. 8a–f). For example, for CD8 cells, *GZMB* upregulation is only part of the “stimulation-biased signature score”, *GZMH* upregulation is part of both scores, and *GZMA* and *GZMM* upregulation is only part of the “ex vivo signature score”. We cross-validated the “ex vivo signature” and “stimulation-biased signature” scores generated based on data from patient GT_3 in patient GT_2 and achieved high linear separability for both scores (Supplementary Fig. 8g, h).

Overall, we could precisely identify the phenotypes of currently and previously activated antigen-reactive PB T cells.

### Matching phenotypes of PB antigen-reactive T cells and the respiratory tract of COVID-19 patients

We next wondered whether we could identify T cells with our antigen-reactive transcriptional signatures in the respiratory tract. Tracheal aspirate (TA) T cells from identical as well as eight additional patients clustered in-between stimulated and unstimulated reactive clonotypes from PB (Supplementary Fig. 9a). TA CD8 T cells showed *IFNG* expression and high cytokine scores, whereas for CD4 T cells neither *IFNG* nor high proliferation scores were detectable (Supplementary Fig. 9b). These results were further corroborated by gene expression analyses of TA T cell clusters (Supplementary Fig. 10). Notably, TA CD4 T cells did not express *ZNF683*, encoding the Tissue-resident memory (TRM) T cell-associated transcription factor Hobit, and expressed little *ITGA1*, encoding the TRM marker CD49a. TA CD8 T cells, in contrast, expressed *ZNF683* and ITGA1 throughout. While these TRM markers differentiated them from antigen-reactive PB CD8 T cells, TA CD8 T cells were overall very similar to reactive PB T cells—especially from the stimulated condition—with high expression of *IFNG*, *PDCD1*, or *CD38* (Supplementary Fig. 10). These data indicated that particularly CD8 T cells from the respiratory tract of COVID-19 patients showed a phenotype that is similar to in vitro activated antigen-reactive CD8 T cells from PB.

To further test this hypothesis and investigate whether this was a feature that is specific for severe disease states, we integrated previously published and publicly available scRNA seq data sets from the respiratory tract of COVID-19 patients with severe disease, mild disease, and healthy controls, from Berlin^47^, Shenzhen^48^, and Chicago^49^ in addition to our own Munich cohort (see “Methods” for further information on data set integration). In total, we analyzed 279,663 cells from 50 patients (including 30,033T cells from 28 patients; Supplementary Fig. 11a). Interestingly, stimulated antigen-reactive T cells from PB were most similar to T cells from nasopharyngeal swabs (NS), which were only present in the Berlin cohort^47^ (Supplementary Fig. 11b). However, in order to compare cell state similarities in a consistent manner and across as many patients and cohorts as possible, we excluded NS samples from further analyses, and only included samples derived from the lower airways (TA and bronchoalveolar lavage fluid, BALF). For some of the severely diseased patients, SARS-CoV-2 transcript was still detectable also in the scRNA seq data (Supplementary Fig. 12a), enabling further categorizations into “severe, virus-positive” and “severe, virus-negative” patients.

**Fig. 4:**
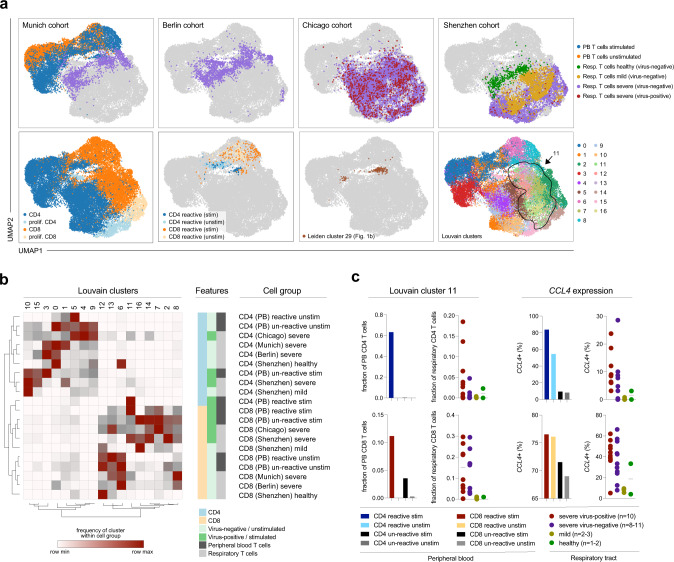
Phenotypic convergence of in vitro stimulated reactive T cells from peripheral blood and ex vivo T cells from the respiratory tract of severely diseased patients. **a** Top panels: UMAPs of previously analyzed peripheral blood (PB) T cells and T cells from tracheal aspirates (TA) of the same (patient GT_3) and additional patients with severe disease (Munich cohort) as well as from bronchoalveolar lavage fluid (BALF) from patients with mild disease, severe disease and healthy donors (Chua et al.^47^—Berlin cohort; Grant et al.^49^—Chicago cohort; Liao et al.^48^—Shenzhen cohort); bottom panels (from left to right): CD4 or CD8 (proliferating or not) cell types, reactive PB T cells from stimulated or unstimulated conditions (see previous figures), IFNG-positive antigen-“reactive” Leiden cluster 29 cells (see Fig. 1b), Louvain clusters (*n* = 30,033 T cells from 28 patients). **b** Hierarchical clustering of cell types and Louvain clusters based on “one minus Pearson” correlations of deviations from baseline compositions for each cell type across Louvain clusters. PB, peripheral blood T cells. Cohort names (Munich, Chicago, Berlin, Shenzhen) refer to respiratory tract T cells. Fractions of CD4 or CD8 T cells in PBMCs or respiratory materials (Supplementary Fig. 12) served as input compositions. **c** Fractions of Louvain cluster 11 or *CCL4* expressing cells among (from left to right and top to bottom) PB CD4 T cells, respiratory CD4 T cells, PB CD8 T cells, or respiratory CD8 T cells. For respiratory T cells, data for individual patients with indicated disease stages are shown (represented by dots; *n* = 2 healthy, *n* = 3 mild, *n* = 11 severe-cold, *n* = 10 severe-hot; patients with <20 cells in Louvain cluster 11 were excluded from analysis).

Stimulated CD4 or CD8 T cells from PB clustered in distinct niches that also contained respiratory T cells from severely diseased patients (Fig. 4a). To map this integrated data set in an unbiased and systematic manner, we applied Louvain clustering and then investigated cell type-dependent enrichments by calculating the share of each cell type within CD8 or CD4 T cells from PB or the respiratory tract across all Louvain clusters (Supplementary Fig. 12b–f). Louvain cluster 11 was enriched for CD8 and CD4 respiratory T cells from severely diseased patients and was at the same time the cluster that encompassed the most reactive PB clonotypes from the stimulation condition (Supplementary Fig. 12b–f). Leiden cluster 29 T cells (Fig. 1b) were also enriched in this area (Fig. 4a). We next analyzed the relative distribution of each cell type across Louvain clusters (Fig. 4b). Hierarchical clustering confirmed the high proximity of respiratory CD8 T cell states to reactive PB CD4 and CD8 T cells from the stimulated condition, which were highly enriched in Louvain cluster 11. Intriguingly, PB CD8 reactive T cells clustered together with respiratory CD8 T cells from severely diseased patients particularly from Chicago and Shenzhen, which in contrast to the Berlin cohort encompassed a substantial number of virus-positive patients very early after entering the ICU. We, therefore, hypothesized that the phenotypic signatures of our stimulated or unperturbed reactive clonotypes could reflect, respectively, “hot” (active virus replication) or “cold” (virus cleared) respiratory tract environments of severely diseased patients, in which, respectively, virus was still detectable or not detectable anymore.

Based on the dominance of stimulated or unperturbed CD4 or CD8 clonotypes, we predicted Louvain clusters to be hot or cold CD4 or CD8 clusters, with cluster 11 being a generally “hot T” cluster and for example, the areas around clusters 5 and 13 reflecting “cold” CD4 and CD8 environments, respectively (Fig. 4a, b). We then analyzed how many respiratory CD4 or CD8 T cells were present in those clusters. Unperturbed T cell signatures of reactive clonotypes were associated with “cold” respiratory environments from severely diseased, but virus-negative patients. In contrast, stimulation-induced T cell signatures of reactive clonotypes were associated with “hot” respiratory environments from severely diseased, virus-positive patients, as most prominently visible for Louvain cluster 11 (Fig. 4c). As an individual gene, *CCL4* was markedly upregulated in respiratory T cells from patients with severe disease, and even more so in virus-positive patients. This is reflected by higher expression in reactive T cells from PB, with particularly pronounced expression in the stimulated condition (see also Supplementary Fig. 8).

Overall, these data indicate that ex vivo stimulation induced a transcriptional state in antigen-reactive T cells from PB which is highly similar to T cells found in the respiratory tract from severely diseased patients with high viral loads (“hot” environments). In contrast, respiratory T cells from severely diseased patients in which no virus is detectable anymore (“cold” environments) showed a phenotype that is similar to PB antigen-reactive T cells when they are not additionally re-stimulated ex vivo. We could thereby interrogate connections between disease stages of individual patients and phenotypic signatures of respiratory T cells, for which currently and previously activated PB T cell subsets provided a framework.

### Modeling intercellular communication between respiratory T cells with antigen-reactive signatures and SARS-CoV-2-positive macrophages

We finally aimed to test how the definition of transcriptional signatures from antigen-reactive T cells after different antigen stimulation conditions can be leveraged across publicly available scRNA seq data sets. To this end, we investigated the intercellular communication between macrophages and respiratory T cells that bear our antigen-reactive signatures. We used scRNA seq data from the “Chicago cohort”^49^, for which a macrophage-T cell circuit has been described. The samples in this cohort of severely diseased patients were acquired 48 h after intubation when the virus was still detectable via scRNA seq. Macrophages in which SARS-CoV-2 transcript was detectable (either after direct infection or after phagocytosis of infected cells) seemed to sense IFNy produced by T cells and in turn, released T cell-targeting cytokines^49^. These macrophages included tissue-resident alveolar macrophages (TRAM) as well as monocyte-derived alveolar macrophages (MoAM). To study the cross-talk between these macrophages and T cells with our antigen-reactive signatures systematically, we applied NicheNet (see “Methods”). The NicheNet algorithm ranks ligands expressed by “sender” cells according to their ability to induce a set of target genes in “receiver” cells. Thereby, the algorithm exploits the transcriptomic signatures of receiver cells, building on prior knowledge on gene regulatory networks from public databases beyond the mere expression of matching ligands and receptors by sender and receiver cells, respectively^50^.

We first investigated which T cell ligands are predicted to induce the differentially expressed genes between SARS-CoV-2 transcript positive and negative macrophages. Compared to SARS-CoV-2-negative TRAM1, SARS-CoV-2-positive TRAM2 highly expressed cytokines such as *CCL2*, *CCL3*, *CCL4*, *CXCL9*, *CXCL10*, and *CXCL11*, as well as molecules that have previously been described to play a role in chemotaxis (e.g., *ICAM1*) and responsiveness to IFNy (e.g., *STAT1*) in the interplay between monocytes and T cells during COVID-19^47^ (Supplementary Fig. 13). *IFNG* and *TNF* were predicted to be the most important ligands expressed by T cells that could induce the transcriptomic changes observed between SARS-CoV-2 transcript positive and negative TRAM. In T cells, the ligands IFNG and TNF were most dominantly expressed in Louvain cluster 11, which also highly expressed the predicted ligands *CCL3* and *CCL4* (Supplementary Fig. 13). The prediction pattern of the additional top-predicted ligands also coincided with the other hot or cold Louvain cluster regions (Fig. 4b). For example, hot CD8 Louvain clusters 2 and 7 (Fig. 4b) stood out through their high expression of the predicted ligand *CCL5* (Supplementary Fig. 13). Further, NicheNet predicted similar T cell ligands inducing the transcriptomic changes between SARS-CoV-2-positive and -negative MoAM (immature MoAM1 vs. mature MoAM2/3, respectively; Supplementary Fig. 14). In summary, unbiased prediction of T cell ligands inducing gene expression changes in SARS-CoV-2 transcript-positive macrophages identified Louvain clusters as the most dominant source of these ligands, which were initially grouped based on the presence of hot or cold T cell signatures and disease stages.

We next wondered whether SARS-CoV-2 transcript carrying macrophages would also signal back to T cells with our antigen-reactive signature in a specific manner. To reflect the “hot” environment of the investigated patients in which the virus was still present, we utilized NicheNet to predict which ligands expressed by Macrophages could induce the genes distinguishing reactive from unreactive T cells from the stimulated condition in CD4 and CD8 T cells. The definition of these target gene sets, including *GZMB*, *IFNG*, *TNF*, *CCL3*, and *CCL4* in stimulated reactive CD4 T cells, led to the prediction of macrophage-derived co-stimulatory ligands such as IL-15, IL-18, CCL4, CCL8, or CXCL9, which have been described to be upregulated in macrophages from the respiratory tract during COVID-19 also in the original studies on the Berlin and Shenzhen cohorts^47,48^ (Supplementary Fig. 15). For CD8 T cells as receivers, NicheNet predicted macrophage-derived co-stimulatory ligands CCL2 and SPP1 (which encodes Osteopontin^51,52^) to drive *CCL3* and *CCL4* expression, as well as IL-15, IL-18, ICAM1, ADAM17, CD80, and CD86 to drive *IFNG*, *TNF*, and *GZMB* expression (Supplementary Fig. 16). Intriguingly, most of the ligands that were predicted on the basis of target genes from our antigen-reactive CD4 and CD8 T cell signatures were preferentially expressed by the very macrophage subtypes in which SARS-CoV-2 transcript was detectable (TRAM2 and MoAM1). Overall, these data indicate a specific ligand-receptor cross-talk between respiratory T cells with antigen-reactive signatures and SARS-CoV-2-positive macrophages.

## Discussion

We here show how barcoding through natural TCR sequences can be used to identify SARS-CoV-2 antigen-reactive T cells after differential stimulation with and without antigen, followed by scRNA seq. Antigen-reactive clonotypes showed consistent transcriptomic shifts upon stimulation, as visible by induction of a distinct cluster in a UMAP projection, expression of generic T cell activation scores along continuous pseudotemporal gradients, and high degrees of inter-correlative gene expression changes. As a single marker, *IFNG* upregulation proved to reliably identify SARS-CoV-2 antigen-reactive CD4 and CD8 T cells. We validated reactive TCRs by transgenic re-expression using CRISPR/Cas9-mediated OTR. Such SARS-CoV-2 reactive TCR-transgenic T cells serve as a potential resource for future immunotherapy of COVID-19 infection. Furthermore, the presented approach represents a generic platform for the identification of antigen-reactive TCRs for a plethora of a priori phenotypically heterogeneous clonotypes at once.

In addition to the identification of antigen-reactive clonotypes per se, “reverse phenotyping” allowed us to investigate the phenotypic profile of antigen-reactive T cells with or without antigenic re-stimulation. This revealed systematic biases induced through re-stimulation and allowed exploration of unperturbed T cell states. By profiling antigen-stimulated CD4 T cells from PB via scRNA seq, it has recently been shown that SARS-CoV-2 antigen-reactive CD4 T cells—particularly in patients with severe disease—show a particularly pronounced cytotoxic profile^10,14,16^ compared to CD4 T cells specific for other viruses^15^. We confirm these findings by demonstrating that CD4 T cells which show major transcriptional shifts upon antigenic stimulation robustly upregulate *IFNG*. However, our “reverse phenotyping” approach also reveals that the classical Th1-ness (as defined by *TBX21* expression) of those cells is overestimated after stimulation, and that—ex vivo—antigen-reactive PB T cells instead show signatures that are far more dominated by *EOMES* and *GATA3* than it appears after stimulation. The role of EOMES in CD4 T cells is less clear compared to other Th lineage defining core transcription factors, but recent evidence suggests that EOMES drives a cytotoxic signature and represses Th17 features^53,54^. Apart from Th1-polarized CD4 cells, antigen-reactive cTfh cells have been reported by several studies^4^. In our patient subset with a severe disease course, we observed *BCL6* and *ICOS* expression in antigen-reactive CD4 clonotypes upon stimulation only. While this does not exclude the possibility that cTfh plays a dominant role in the adaptive immune response to SARS-CoV-2, these findings provide examples of the phenotypic biases introduced by antigen stimulation prior to phenotyping.

Both CD4 and CD8 antigen-reactive T cells showed TEM/T cell effector phenotypes with less expression of co-inhibitory molecules and markers of terminal differentiation than would be expected based on the phenotypes after stimulation. However, at the same time, antigen-reactive CD4 and CD8 T cells were characterized by a more cytotoxic and differentiated profile in comparison to antigen-unreactive T cells when stimulation biases are accounted for. Thereby, reverse phenotyping allowed us to define more accurately the actual ex vivo phenotypic profiles of SARS-CoV-2 antigen-reactive T cells. We performed reverse phenotyping on T cells only from PB, in a limited number of patients with severe disease, and focused on T cell reactivity to virus spike antigens. More comprehensive experimental analyses of SARS-CoV-2 antigen-reactive T cells are needed—including different time points and disease severities—to more comprehensively map the ex vivo phenotypic profile of SARS-CoV-2 specific T cells. Also, investigation of additional antigen groups (including the whole SARS-CoV-2 proteome) and specific epitopes (to also account for potential underrepresentation in the 15mer peptide mix that was used for this study) will be of importance for future studies, explicitly including antigenic changes as present in SARS-CoV-2 variants of concern. Here, we provide proof of concept that “reverse phenotyping” can be used for that purpose.

Publicly available data sets—particularly those that are bundled in a coherent manner as within the Human Cell Atlas initiative—represent a vast resource of deeply profiled immune cells from many different patients and disease contexts. However, in these data sets often no dedicated antigen-specific T cell analyses have been performed. In T cells from the respiratory system of identical as well as further patients—including patients from independent reference cohorts—we detected transcriptional signatures that overlapped with those we defined based on antigen-reactive T cells from PB. In vitro stimulated reactive T cells thereby mirrored “hot” cell states from virus-positive patients with severe disease, whereas unperturbed T cell states reflected “cold” cell states from virus-negative patients with severe and mild disease. Interaction of T cells with viral antigens at the site of infection could explain these findings. In the future, it will be exciting to further investigate the role of SARS-CoV-2 antigen-reactive T cells at the site of infection as well as in PB, e.g., by using TCR sequences to precisely define ontogenic relationships.

As a tool for T cell immunology, scRNA seq after differential antigen stimulation opens up avenues for the identification and characterization of antigen-reactive T cells by exposing gradients of antigen reactivity and reverse phenotyping. It thereby contributes to a deep understanding of the adaptive immunity induced by a viral infection, which will be pivotal to guide the enhanced and accelerated development of therapies and vaccines for emerging pathogens such as SARS-CoV-2.

## Methods

### Munich cohort patients

#### Clinical information and material

All Munich cohort patients were PCR-confirmed SARS-CoV-2 positive, admitted to the ICU in the University Hospital of the Ludwig-Maximillian’s University, Munich (*n* = 5), or the Asklepios Lung Clinic Munich-Gauting, Gauting (*n* = 4), for treatment of severe COVID-19 requiring invasive, mechanical ventilation. For further clinical information see Supplementary Data 1. PBMCs and TA samples were taken at the end of April 2020.

#### Consent

Written informed consent was obtained from the donors or their caregivers, usage of the blood samples was approved according to national law by the local Institutional Review Board (Ethikkommission der Medizinischen Fakultät der Ludwigs-Maximilian-Universität München; vote IDs 19-629, 19-630, and 20-259) and/or samples were used according to legal provisions defined by the German Infection Protection Act (IfSG).

### T cells from PBMCs

#### Cell culture

PBMC were isolated from EDTA whole blood by gradient density centrifugation according to the manufacturer’s instructions (Biocoll, Biochrom) and frozen in FCS + 10% DMSO (Merck) for liquid nitrogen storage. After freezing–thawing procedure, T cells were cultured in RPMI 1640 (Gibco) supplemented with 5% human serum, 0.025% l-glutamine, 0.1% HEPES, 0.001% gentamycin, and 0.002% streptomycin (hereafter RPMI-HS).

#### Antigen-specific stimulation and flow cytometry-assisted cell sorting prior to scRNA seq

PBMCs were stimulated with 0.6 nmol of SARS-CoV-2 spike protein–peptide mix (PepTivator^®^SARS-CoV-2 Prot_S, Miltenyi). For the unstimulated condition, PBMCs were only cultured in RPMI-HS. After stimulation for 4 h at 37 °C, surface staining was conducted for 20 min with the following fluorochrome-conjugated antibodies: CD3-APC (1:200), CD4-PE (1:100), CD56-FITC (1:100), CD8-eFluor450 (1:200) (Life Technologies, 17-0038-42, MHCD0404, 11-0566-42, 48-0086-42,) and CD19-ECD (1:100) (Beckman Coulter, A07770). Propidium iodide (Invitrogen) was used for live/dead discrimination. Flow sorting of CD4+ and CD8+ cells from the stimulated and unstimulated condition was conducted on a MoFlo Astrios EQ (Beckman Coulter) under biosafety level 3. For a representative gating strategy, see Supplementary Fig. 17a.

#### TCR DNA template design and CRISPR–Cas9 mediated TCR KI

DNA constructs for CRISPR–Cas-9-mediated HDR at TRAC locus were designed in silico with the following structure: 5′ homology arm (300–400 base pairs (bp)), P2A, TCR-β (including mTRBC with additional cysteine bridge^55^), T2A, TCR-α (including mTRAC with additional cysteine bridge), bGHpA tail, 3′ homology arm (300–400 bp). All HDR DNA template sequences were synthesized by Twist Bioscience. CRISPR-Cas9-mediated TCR KO and KI were performed as previously described^28^ on isolated PBMCs from donor whole blood. In short, bulk PBMCs were activated for two days in RPMI with CD3/CD28 Expamer (provided by Juno Therapeutics), 300 IU/ml recombinant human IL-2, 5 ng/ml recombinant human IL 7 (Peprotech, reference #200-07), and 5 ng/ml IL-15. Expamer stimulus was removed by incubation with 1 mM d-biotin (Sigma, reference #D1411-1G). Totally, 1 × 10^6^ cells were electroporated (pulse code EH100) with Cas9 ribonucleoprotein and DNA templates in 20 µl Nucleofector Solution P3 (Lonza, reference #V4SP 3096) with a 4D Nucleofector X unit (Lonza). After electroporation, cells were cultured in RPMI supplemented with 180 IU/ml IL-2 until a first fluorescence-activated cell sorting (FACS) analysis on day five after editing. Sequences of experimentally tested TCRs can be found in Supplementary Data 10. Sequences of primers to amplify targeting constructs can be found in Supplementary Data 13.

#### Antigen-specific activation of TCR-engineered T cells and intracellular cytokine staining

PBMCs for autologous peptide pulsing were isolated and cultured in RPMI-HS with 50 IU/ml human IL-2 (Peprotech). On the day of antigen-specific activation of CRISPR–Cas9-engineered T cells, autologous PBMCs were pulsed with 10 µg/ml SARS-CoV-2 spike protein–peptide mix (PepTivator^®^SARS-CoV-2 Prot_S, Miltenyi) for 2 h at RT and gentle agitation. After peptide pulsing, the excess peptide was removed by washing, and PBMCs were cocultured with CRISPR–Cas9-engineered T cells in 1:1 and 1:3 effector:target ratio and Golgi-Plug (BD Biosciences) for 4 h at 37 °C. Surface marker antibody staining for CD3-BV421 (1:100) (BD Biosciences, 563797), CD8-PE (1:200) (Life Technologies, 12-0086-42) and murine TCR β-chain-APC/Fire750 (1:50) (Biolegend, 109246) were followed by permeabilization using Cytofix/Cytoperm (BD Biosciences) and intracellular staining of IL-2-APC (1:25) (BD Pharmingen, 341116). Live/dead discrimination was performed by using ethidium-monoazide-bromide (Invitrogen). FACS samples were acquired on a Cytoflex (S) flow cytometer (Beckman Coulter). For a representative gating strategy, see Supplementary Fig. 17b.

### Single-cell RNA sequencing

#### PB T cell processing

CD3+ CD4+ and CD8^+^ T cells were sorted by flow cytometry, centrifuged and the supernatant was carefully removed. Cells were resuspended in the Mastermix + 37.8 µl of water before 70 µl of the cell suspension were transferred to the chip (step 1.1 and 1.2 of the original protocol). After each step, the integrity of the pellet was checked under the microscope to ensure that all cells are loaded onto the chip. From here on, 10x experiments have been performed according to the manufacturer’s protocol (Chromium next GEM Single Cell VDJ V1.1, Rev D). Quality control has been performed with a high sensitivity DNA Kit (Agilent #5067-4626) on a Bioanalyzer 2100 as recommended in the protocol and libraries were quantified with the Qubit dsDNA hs assay kit (life technologies #Q32851). All steps have been performed using RPT filter tips (Starlab #S1181-3710) and DNA LoBind tubes (Sigma #EP0030108051, #EP0030108078, and #EP0030124359).

#### TA processing

TAs were digested with 4 ml dispase (50 units/ml) (Corning, #354235) and 25 µl DNase (30 μg/ml) (Qiagen, #79254) at 37 °C for 10 min with occasional shaking. The digestion was then stopped with 10 ml of ice-cold 10% FCS/PBS. To obtain single-cell suspensions, the digestion mix was passed through a 70 µm cell strainer. Red blood cell lysis was performed only when necessary by incubating the cells with 3 ml RBL buffer at RT for 1 min. The cells were counted, diluted to 1000 cells/µl, and loaded on the 10× Chromium Next GEM Chip G with a targeted cell recovery of 10,000. The following steps were completed according to the manufacturer’s protocol (Chromium Next GEM Single Cell 3ʹ Reagent Kits v3.1).

#### Next-generation sequencing

Libraries have been pooled according to their minimal required read counts (35,000 or 50,000 reads/cell for 3′ gene expression libraries, 20,000 reads/cell for 5′ gene expression libraries, and 5000 reads/cell for TCR libraries). Illumina paired-end sequencing was performed with 150 or 200 (3′ gene expression) and 100 cycles (5′ gene expression and TCR libraries) on a NovaSeq 6000.

### Single-cell RNA sequencing data analysis

#### Data processing

After sequencing, the processing of next-generation sequencing reads of the scRNA-seq data was performed using CellRanger version 3.1.0 (10× Genomics) with a customized reference genome. To account for transcripts originating from the virus, the genes of the SARS-CoV-2 genome (NCBI Reference Sequence: NC_045512.2) were manually added to the hg38 (GRCh38.99) human reference genome’s GTF files. Negative strands of the viral genes were additionally added. The genome was indexed via CellRanger’s “mkref” command.

#### Initial unsupervised analysis of PB T cells

We performed unsupervised analysis for multiple subsets of the data with scanpy^56^ (v1.6.0): Both patients, patient GT_3 only, patient GT_2 only, stimulated or unstimulated condition only for both patient GT_3 and GT_2, separately. In all cases, we first filtered genes that were expressed in at least 10 cells, scaled cell-wise expression vectors to a total count of 10,000, and logp1-transformed the data and selected highly variable genes (flavor = ”seurat). We then performed a principal component analysis, computing 50 components, based on which we computed a k-nearest neighbor graph (*k* = 50). We then computed UMAPs^57^ and Leiden or Louvain clusterings^58^. The complete code for this analysis is reported in the Source Data file.

#### Cell type assignment of PB T cells

CD4 and CD8 expressing cells did not clearly separate in Leiden groups. Therefore, we chose a two-staged cell type assignment process: First, we assigned Leiden groups to either CD4+ or CD8+ T cells based on their relative mean expression in the group. Secondly, we re-assigned cells from clonotypes (see also: Clonotype analysis), which contained both putative CD4+ and CD8+ T cells, to the major cell type found in this clonotype.

#### Cell state assignment of PB T cells

We re-clustered CD4 and CD8 cells in the unstimulated condition separately using the Leiden algorithm to identify cellular states at a higher resolution than cell types. For this purpose, we repeated the unsupervised analysis as described above on this subset of cells. Throughout the manuscript, these clusters are ordered by similarity in heatmaps and dot plots. This ordering is derived from a hierarchical clustering based on all genes with mean expression larger than 0.5 in the selected subset.

#### Clonotype assignment of PB T cells

We performed TCR sequence analysis with scirpy^59^ (v0.4). We defined clonotypes based as groups of cells with identical nucleotide CDR3 sequences of the primary TRA and TRB chains. We performed clonotype assignment for both patients separately, but across both conditions, stimulated and unstimulated, together. Clonotype sequences can be found in Supplementary Data 11 and 12. The complete code for this analysis is reported in the Source Data file.

#### Pseudotime analysis of PB T cells

We computed pseudotime-based scores by computing diffusion pseudotime^60^ with respect to a cell in the reactive T cell cluster in patient GT_3. This root T cell was selected based on its extremal position in the UMAP, at the tip of the reactive cluster. The nearest-neighbor graph underlying the pseudotime computation was derived as described in section initial unsupervised analysis. The complete code for this analysis is reported in the Source Data file.

#### GATA3 and TBX21 correlation

We investigated *GATA3* and *TBX21* correlation in the CD4 cells with two techniques: Firstly, we computed their log-normalized expression covariance. Secondly, we computed expected rates of *GATA3*− *TBX21*−, *GATA3*+ *TBX21*−, *GATA3*− *TBX21*+ and *GATA3*+ *TBX21*+ cells based on the marginal frequency of *GATA3*+ and *TBX21*+ cells and compared these to the observed frequencies of single- and double-positives. Here, positive cells were defined as cells with any detected UMIs of the selected gene. The complete code for this analysis is reported in the Source Data file.

#### Differential expression analysis of PB T cells

We performed differential expression analysis with diffxpy (v.0.7.4): We used Welch’s *t* test on log-normalized UMIs to compare expression differences between two groups of cells. We labeled genes as differentially expressed if they had a Benjamini–Hochberg corrected *p* value of less than 0.01 and a mean expression of at least 0.5.

#### Tissue residency score of PB T cells

We computed expression z-scores for the following genes associated with tissue residency^61^: *CA10*, *CRTAM*, *CX3CR1*, *CXCL13*, *CXCR6*, *DUSP6*, *FAM65B*, *IL2*, *IL10*, *IL23R*, *ITGA1*, *ITGAE*, *KCNK5*, *KLF2*, *KLF3*, *KRT72*, *KRT73*, *NPDC1*, *PDCD1*, *PTGDS*, *RAP1GAP2*, *RGS1*, *S1PR1*, *SBK1*, *SELL*, *SOX13*, *STK38*, *TSPAN18*, *TTC16*, and *TTYH2*. We aggregated these cell- and gene-wise *z*-scores to a cell-wise tissue residency score by taking the mean across all gene-wise *z*-scores.

#### Unsupervised analysis of TA data

The count matrices output by CellRanger were again analyzed using Scanpy^56^ (v.1.6.0). For barcode filtering, we excluded barcodes with less than 200 detected genes. A high proportion (>10%) of transcript counts derived from mitochondria-encoded genes may indicate low cell quality, and we removed these unqualified cells from downstream analysis. The number of unique molecular identifiers (UMIs) was explored via patient-wise violin plots, and individual upper cut-offs were applied ranging from 5000 to 80,000 UMI counts. Genes were only considered if they were expressed in at least three cells in the data set. As we observed a certain degree of ambient RNA bias, we applied SoupX version 1.3.6^62^ to lessen this effect with mostly default parameters, setting the contamination fraction manually to 0.3.

The expression matrix was normalized using scran’s normalization approach^63^, in which size factors are calculated and used to scale the counts in each cell. The data were then log1p-transformed and the top 4000 variable genes were selected for each patient individually (flavor = ”seurat). Genes that were listed as a variable in at least 3 patients (5984 genes after removing cell cycle genes) were used as basis for the principal component analysis. BBKNN^64^ was employed to construct a batch-corrected neighborhood graph using the first 20 components and individual patient labels as batch keys. Unsupervised clustering was performed with Louvain at resolution three and cells coarsely annotated based on the manual exploration of known marker genes. As T cell subtypes were of particular interest, we subdivided the data set to *CD3E* high cluster and refined the annotation after re-clustering and assessing of mean expression of *CD8A*, *CD8B*, and *CD4* in the respective cluster.

### Computational data integration

#### Computational data integration—Munich TA and PBMC

In order to compare the results from the PBMC data set with ex vivo patient samples, the normalized, log1p-transformed matrices of both objects after initial filtering were concatenated. The variable gene selection was executed anew with more stringent parameters on this combined object. The top 800 genes (flavor = “cell_ranger”) were selected for both data sets separately, resulting in 183 variable genes labeled as such in both calculations. Principal component analysis (PCA) and the neighborhood graph were re-calculated using 50 components and 15 neighbors. The cell type annotation established on the separate objects was retained.

#### Computational data integration—includes recent Covid-19 related BALF data sets

To test the generalizability of our results on independent cohorts and in order to include varying levels of severity and healthy controls in our analysis, we combined our single-cell data (Munich cohort) with recently published Covid-19 related data sets. The raw count matrices and metadata from the Shenzhen^48^, Chicago^49^, and Berlin^47^ cohorts were downloaded and pre-processed separately with Scanpy (v1.6.0). Briefly, for each of the data sets cells that were present in the final provided object were retained. Additionally, we re-calculated the number of counts and genes per cell and applied the following thresholds: only cells with more than 200 genes and less than 15% mitochondrial transcripts were kept. We assessed the number of UMIs with sample-wise violin plots like the number of detected transcripts varied across the cohorts. Following upper thresholds were chosen: 6000 for the Shenzhen cohort, 30,000 for the Chicago cohort, and 200,000 for the Berlin cohort.

The expression matrices of each data set were normalized and log1p-transformed separately as described above for the Munich TAs. For a first lighter batch correction we defined the list of variable genes in a way that decreased cohort-specific effect as follows. First, highly variable genes were selected (flavor = ”cell_ranger”) for each sample separately, returning the top 4,000 variable genes per individual. For the Munich, Shenzhen, and Chicago cohorts, we considered a gene as highly variable if it is labeled as such in at least three patients of the respective cohort. As the Berlin cohort contained far more patients, we increased the threshold to six patients.

After quality control the ambient RNA contamination was assessed and removed using SoupX^62^ with mostly default parameters, setting the contamination fraction manually to 0.3.

Next, the preprocessed count matrices from the individual data sets were merged and the highly variable genes were set to the intersection of the four cohort-wise lists. After excluding genes associated with the cell cycle, 1370 variable genes remained, which were used as input to PCA (*n* = 50).

The published cell type assignments from each cohort were used further, the labels were harmonized as some annotations for the same cell type showed slight changes in spelling. Cells with unclear annotation were removed from the analysis (“Doublets”, “hybrid”, “unknown_epithelial”, “IRC”).

For visualization of the concatenated data sets, a UMAP and a batch-corrected neighborhood graph was constructed via BBKNN^64^ using ten neighbors within each batch with *cohort* used as a batch key.

#### Computational data integration—T cell subtypes

The cells with the following annotations were used to subset to a T cell only data set: “CD8 T, CCR7+ T, Proliferating T, Treg” (Shenzhen cohort^48^), “CD4 T cells, CD8 T cells, Proliferating CD4 T cells, Proliferating CD8 T cells, Tregs” (Chicago cohort^49^) and “CTL, Treg” (Berlin cohort^47^). For the Berlin cohort, only T cells from BALF samples were used further, cells originating from nasal swabs are only shown in the data set encompassing all cell types. The cell type annotation for the Shenzhen and Berlin cohort did not distinguish between CD4 and CD8 T cells and was therefore refined in an additional step. After re-calculating of the principal components and Louvain clustering, manual examination of CD8A, CD8B, CD4A, CD3E expression enabled a more fine-grained annotation of the subtypes comparable with the Munich cohort annotation. Using the variable gene list established on the full integrated data set, the PCA was re-calculated and the batch corrected neighborhood graph re-constructed (n_pcs = 50, neighbors_within_batch = 20, batch_key = “data_set”).

#### Hierarchical clustering of cell-type frequencies across Louvain clusters

Hierarchical clustering of cell-type frequencies across Louvain clusters was performed with Morpheus (https://software.broadinstitute.org/morpheus). Both Louvain clusters and cell types were clustered based on ‘one minus Pearson’ correlations.

#### NicheNet analysis

We performed NicheNet analysis on the scRNA seq reference cohort from Chicago using the R (version 3.6.3) packages nichenetr^50^ (version 1.0) and Seurat^65^ (version 3.2): To analyze cell-cell communication between Macrophages and CD4 or CD8 T cells respectively, we adopted the cell type annotations from the Chicago dataset and defined the five distinct Macrophage subpopulations contained therein (MoAM1, MoAM2, MoAM3, TRAM1, and TRAM2) as sender cell types and all CD4 or CD8 T cells as receiver cell types, respectively. We then defined the genes comprising the different “ex vivo signature scores” for CD4 and CD8 T cells as gene sets of interest for CD4 and CD8 T cells, respectively.

To analyze cell–cell communication between individual T cell subsets and TRAM or MoAM, respectively, we used the resulting 17 Louvain clusters from the integrated analysis as sender cell types and all TRAMs or MoAMs (see original annotation from the Chicago reference cohort) as receiver cell types. To define a gene set of interest, we performed differential expression testing at the gene level between TRAM1 vs. TRAM2 and immature MoAMs (MoAM1) vs. mature MoAMs (MoAM2 and MoAM3) using a Wilcoxon Rank Sum test with Seurat’s FindMarkers() function. We only included genes with a log-fold change of >0.25, if they were expressed in >10% of all cells, and if their Holm-Bonferroni adjusted *p* value was <0.05.

For all NicheNet analyses, we identified a list of potentially active ligands expressed by sender cell types and ranked them according to their ability (Pearson’s correlation coefficient) to predict the gene set of interest. We then selected the 12 top-ranked ones for subsequent analysis. The complete code for this analysis is reported in the Source Data file.

### Software

Flow-cytometric data were analyzed with FlowJo v10.4.2. For visualization and some statistical analysis, GraphPadPrism (v8.4.3) was used. We performed analysis of scRNA seq data with CellRanger version 3.1.0 (10× Genomics), scanpy (v1.6.0), scirpy (v0.4), diffxpy (v.0.7.4), and SoupX (v1.3.6). Some hierarchical clustering analyes were performed with Morpheus (https://software.broadinstitute.org/morpheus; no version number indicated; version used as of September 2020). NicheNet analyses were performed with R (version 3.6.3) packages nichenetr (version 1.0) and Seurat (version 3.2). For code, see the Source Data file.

### Reporting summary

Further information on research design is available in the Nature Research Reporting Summary linked to this article.

## Supplementary information

Supplementary Information

Description of Additional Supplementary Files

Supplementary Data 1

Supplementary Data 2

Supplementary Data 3

Supplementary Data 4

Supplementary Data 5

Supplementary Data 6

Supplementary Data 7

Supplementary Data 8

Supplementary Data 9

Supplementary Data 10

Supplementary Data 11

Supplementary Data 12

Supplementary Data 13

Reporting summary

## Data Availability

All data generated or analyzed during this study are included in this article, its supplementary information files (Source Data file) and are available at NCBI GEO under the accession number GSE171037. Additional raw data are available from the corresponding authors upon reasonable request. The three independent reference cohorts were accessed as follows: “Shenzhen”: Single-cell landscape of bronchoalveolar immune cells in patients with COVID-19 [Liao et al.^48^]: All data used in this study, including scRNA-seq and scTCR-seq raw data, filtered expression matrix, and scTCR-seq contig annotation that support the findings of this study can be accessed in GEO under the accession number GSE145926. “Chicago”: Alveolitis in severe SARS-CoV-2 pneumonia is driven by self-sustaining circuits between infected alveolar macrophages and T cells [Grant et al.^49^] Single-cell RNA-seq: Counts tables and integrated objects are available through GEO with accession number GSE155249. “Berlin”: COVID-19 severity correlates with airway epithelium-immune cell interactions identified by single-cell analysis [Chua et al.^47^] In addition, count and metadata tables containing patient identification, sex, age, cell type, and quality control metrics for each cell are available at FigShare: 10.6084/m9.figshare.12436517. https://figshare.com/articles/COVID-19_severity_correlates_with_airway_epithelium-immune_cell_interactions_identified_by_single-cell_analysis/12436517 Source data are provided with this paper.

## Source data

Source Data

## Code availability

For scRNA seq data analysis algorithms see the “Methods” section and jupyter notebooks in the Source Data file. Additional information is available from the corresponding authors upon reasonable request.
