# Supplementary material for: Single-cell RNA sequencing reveals ex vivo signatures of SARS-CoV-2-reactive T cells through ‘reverse phenotyping’
Source: Nat Commun. 2021 Jul 26;12:4515. doi: 10.1038/s41467-021-24730-4 (PMC8313584; doi:10.1038/s41467-021-24730-4)
Supplement: Supplementary file 1 — Supplementary Information [file 41467_2021_24730_MOESM1_ESM.pdf]

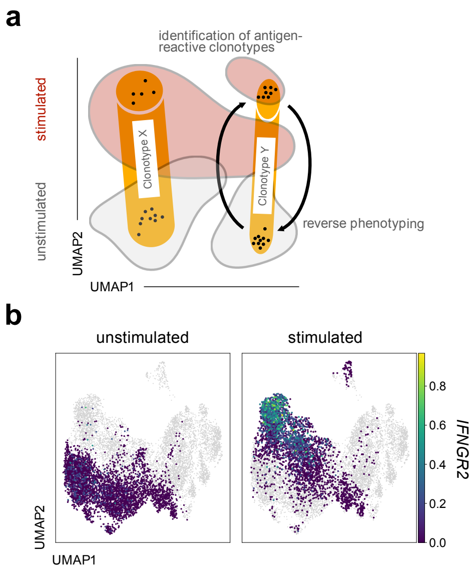

Extended Data Figure 1

Supplementary Figure 1. Scheme of experimental approach and *IFGNR2* expression in CD4 T cells. a, Scheme of experimental approach to use scRNA seq of differentially stimulated cells and TCRs as natural barcodes for cells belonging to shared clonotypes for identification of antigen-reactive clonotypes and reverse phenotyping. **b,** UMAP of all T cells (n=11,460 cells in total), with CD4 T cells from the stimulated and unstimulated condition, highlighted separately with log-normalized *IFGNR2* expression superimposed. Data are shown for patient GT_3.

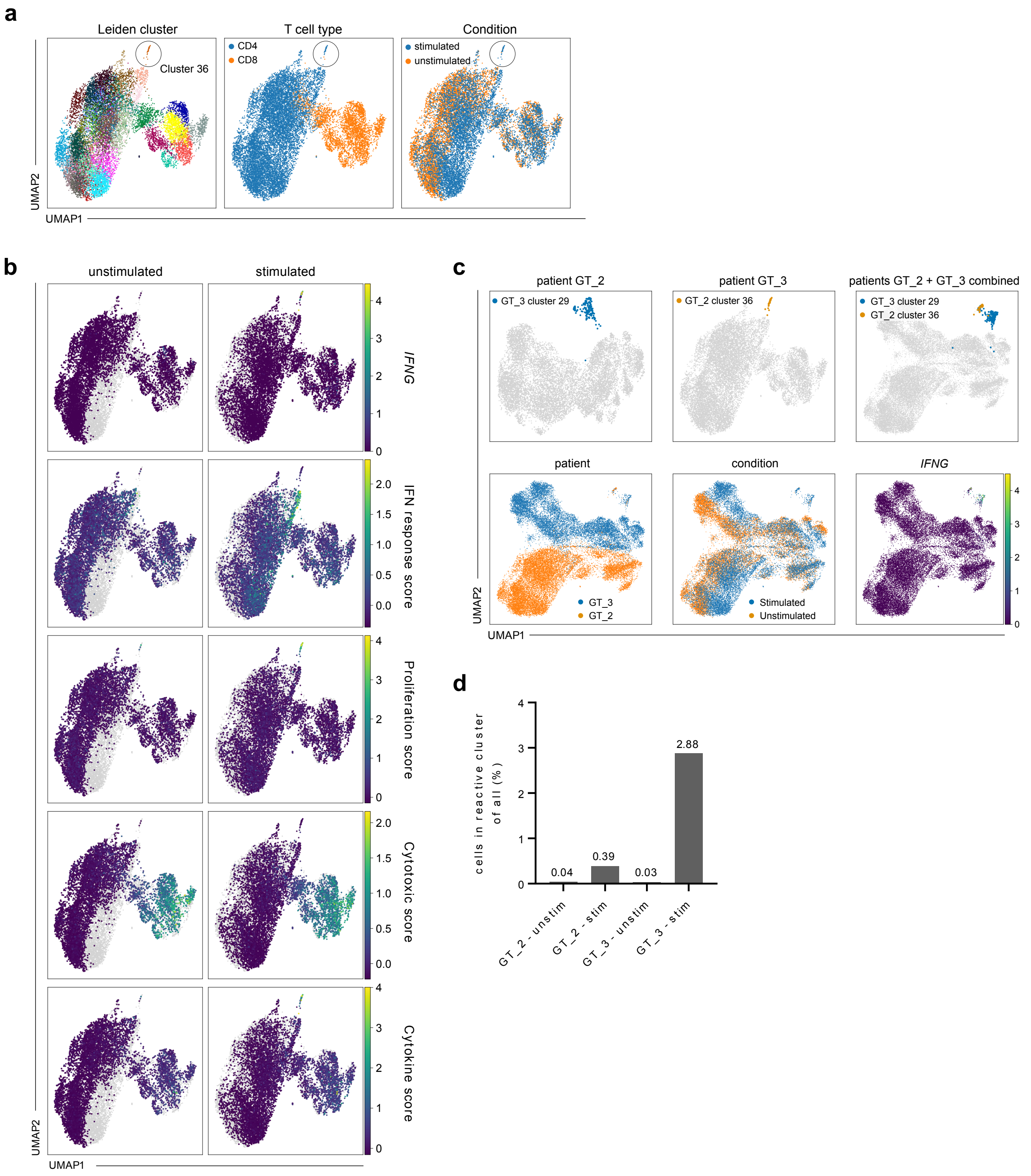

Extended Data Figure 2

Supplementary Figure 2. Unsupervised analysis of PBMC of patient GT_2. a, b, UMAPs of all T cells of patient GT_2 (n=15,189 cells in total) with Leiden cluster, cell type and condition superimposed (**a**) and log-normalized *IFNG* expression and selected scores superimposed (**b**). **c,** Individual UMAPs from patient GT_3, GT_2 as well as integrated UMAP from both patients with stimulation-reactive Leiden clusters, patient ID, condition and *IFNG* expression highlighted. **d,** Percentage of cells in 'reactive clusters' (cluster 29 for patient GT_3 and cluster 36 for patient GT_2) with or without stimulation.

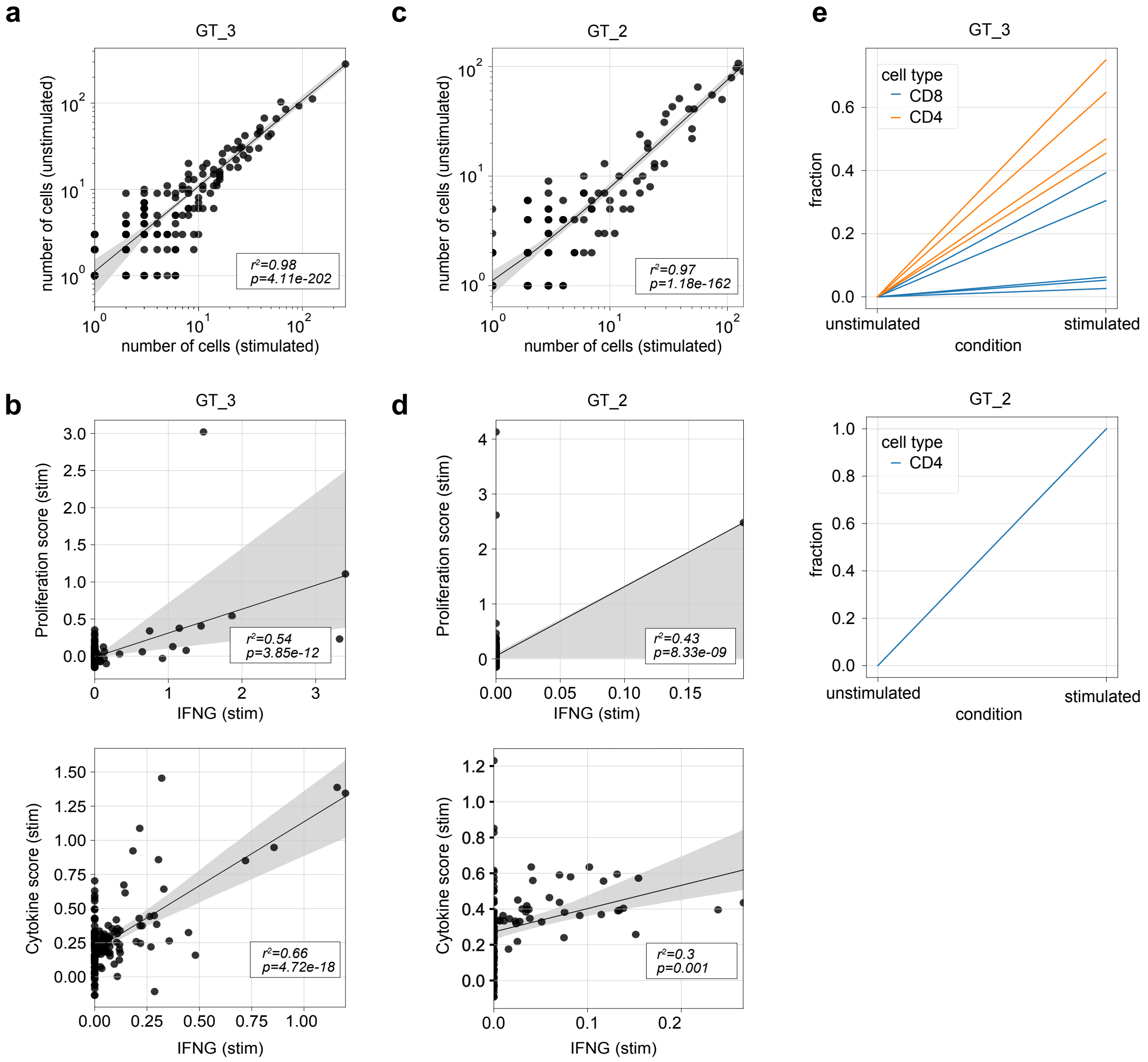

Extended Data Figure 3

Supplementary Figure 3. Clonotype summary statistics. a, Scatter plot showing number of cells of clonotype in stimulated condition versus number of cells in the unstimulated condition (n=279 clonotypes). **b,** Mean log-normalized *IFNG* expression versus mean proliferation score in CD4 T cells (top) or versus mean cytokine score in CD8 T cells (bottom) in stimulated condition by clonotype. Data are shown for patient GT_3 (n=279 clonotypes). **c, d,** As in a and b, only for patient GT_2 (n=278 clonotypes). r^2 and (two-tailed) p-values were calculated after Pearson correlation analysis; grey area indicates 95% confidence interval of linear fit (a-d). **e,** Fraction of cells per clonotype in the reactive cluster (among clonotypes that are found in the reactive cluster), for each condition, cell type and patient.

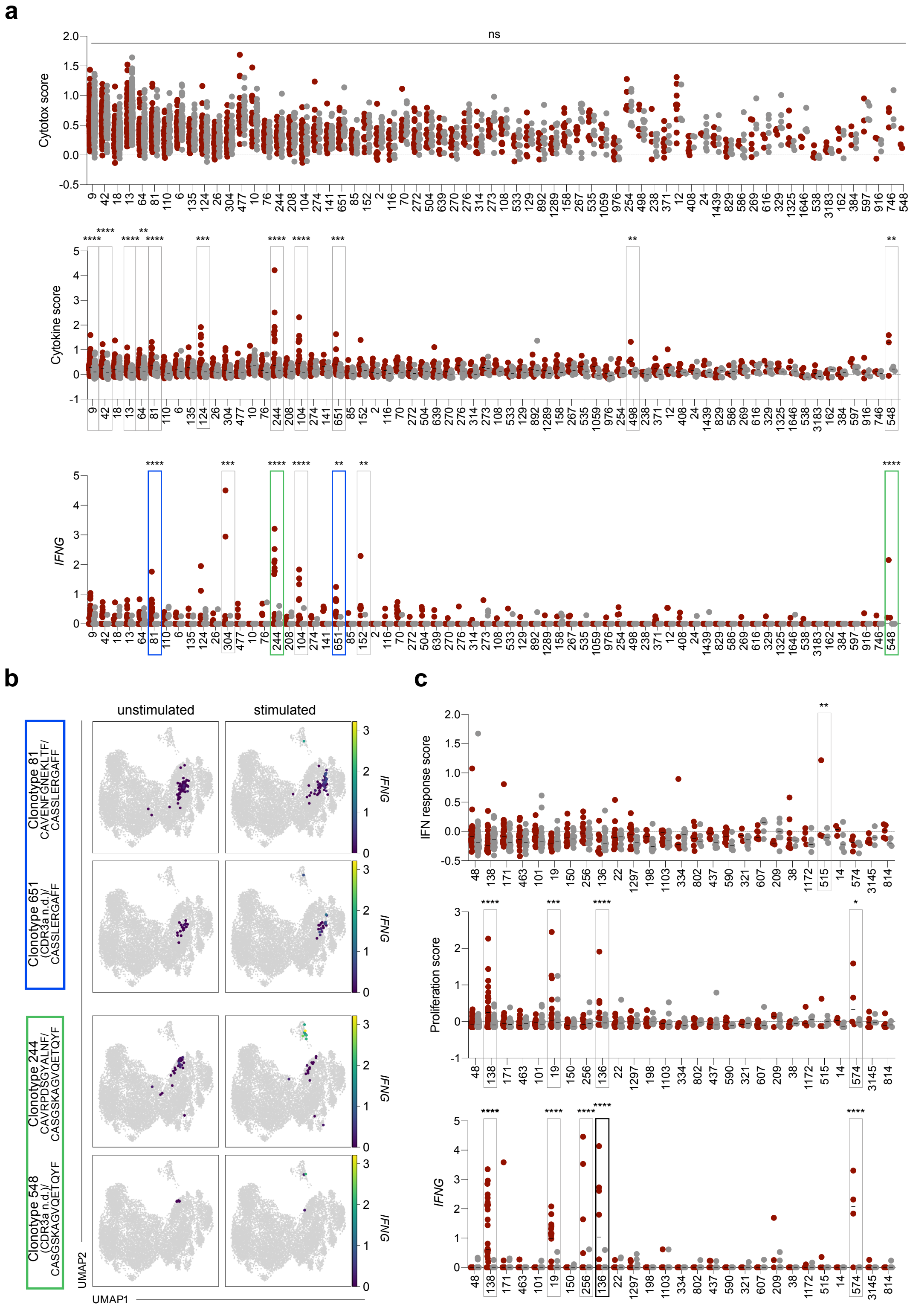

Extended Data Figure 4

Supplementary Figure 4. Clonotype-wise activation statistics. a, Cell-wise cytotoxic score, cytokine score, and log-normalized *IFNG* expression by clonotype in CD8 T cells. Cytokine score: $p < 0.0001$ for clones 9, 42, 13, 81, 124, 244, 104; $p = 0.089$ for clone 64; $p = 0.0004$ for clone 651; $p = 0.0083$ for clone 498; $p = 0.005$ for clone 548. *IFNG* expression: $p < 0.0001$ for clones 81, 244, 104 and 548, $p = 0.0007$ for clone 304, $p = 0.0011$ for clone 651, $p = 0.0037$ for clone 152. **b,** UMAPs of all T cells with cells from selected CD8 clonotypes highlighted with log-normalized *IFNG* expression superimposed (n=11,460 cells in total). **c,** Cell-wise IFN response score, proliferation score, and log-normalized *IFNG* expression by clonotype in CD4 T cells. IFN response score: $p = 0.0079$ for clone 515; proliferation score: $p < 0.0001$ for clones 138, 136; $p = 0.0009$ for clone 19; $p = 0.0103$ for clone 574; *IFNG* expression: $p < 0.0001$ for clones 138, 19, 256 and 574. In contrast to Fig. 2a-b, additionally clonotypes that were defined through a single TCR chain were incorporated into the analyses; as in Fig. 2a-b, only clonotypes with at least three cells in each condition were considered (a-c); reactive single-chain clonotypes are highlighted in blue/green (a) and black (c); reactive partner clonotypes based on shared CDR3a sequences are shown in corresponding colors (a). Each dot represents one cell and lines indicate median. Exact cell numbers per clonotype can be found in Supplementary Data 11 (a,c). Data are shown for patient GT_3. Statistical analysis by two-way ANOVA (**** each for treatment effect and clonotype distribution) followed by Sidak's multiple comparisons test * $p < 0.05$, ** $p < 0.01$, *** $p < 0.001$, **** $p < 0.0001$ (**a, c**).

a

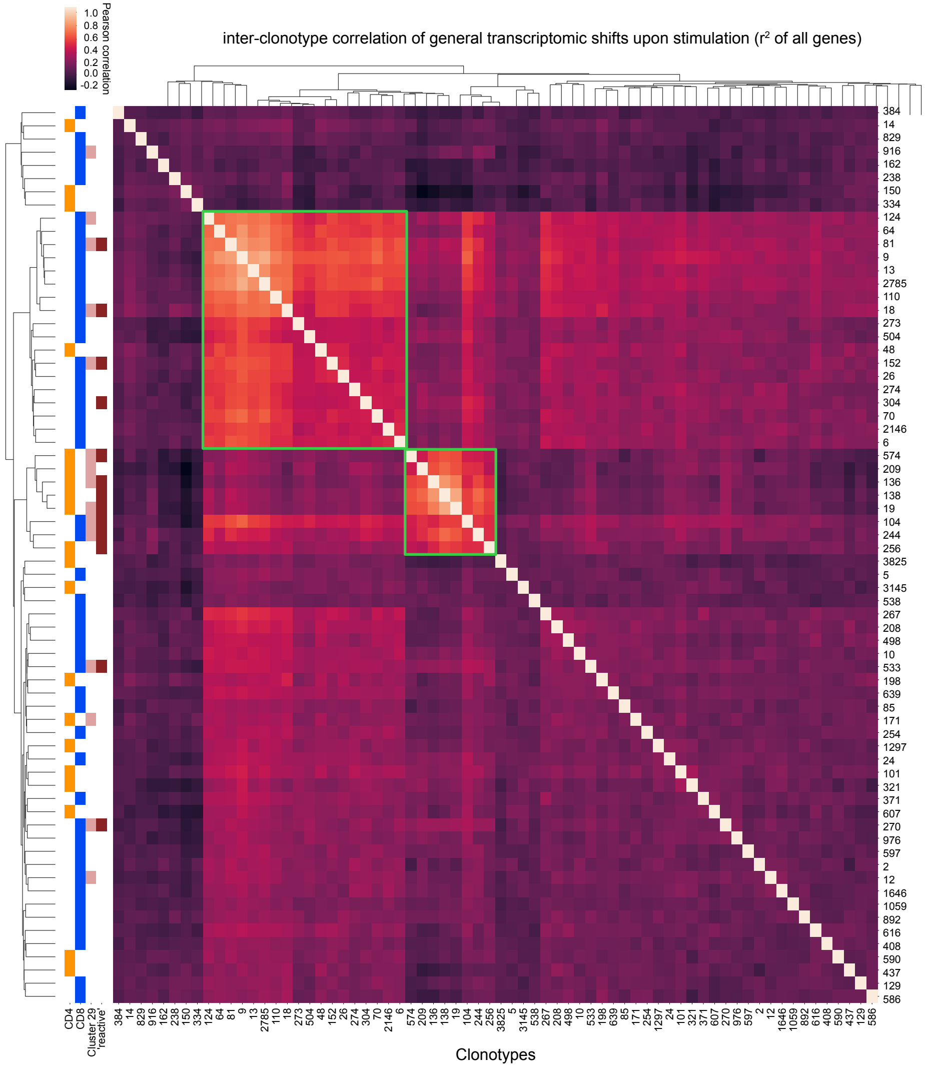

b

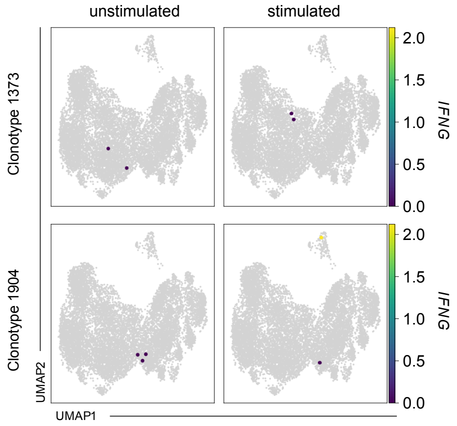

Extended Data Figure 5

Supplementary Figure 5. Inter-clonotype correlation of general transcriptomic shifts upon stimulation and stimulation-induced shifts for two small clonotypes. a, Pearson correlation of stimulation vectors per clonotype. On the left, clonotypes are assigned to CD4+ T cells (CD4), CD8+ T cells (CD8), to having cells in the IFNG+ reactive cluster 29 (Fig. 1b) and as to whether the clonotype was identified as 'reactive' (Fig. 2a-b). Stimulation vectors reflect the difference between mean log-normalized counts of the 2000 highest expressed genes between stimulated and unstimulated condition for each clonotype that had at least three cells in each condition. Clonotypes were clustered using hierarchical clustering. **b,** *IFNG* expression in unstimulated or stimulated T cells for indicated CD4 clonotypes. For each clonotype, cells belonging to that clonotype are shown in an individual panel pair (cells from unstimulated condition in left panels, cells from stimulated condition in right panels), while cells not belonging to that clonotype are shown in grey (n=11,460 cells in total).

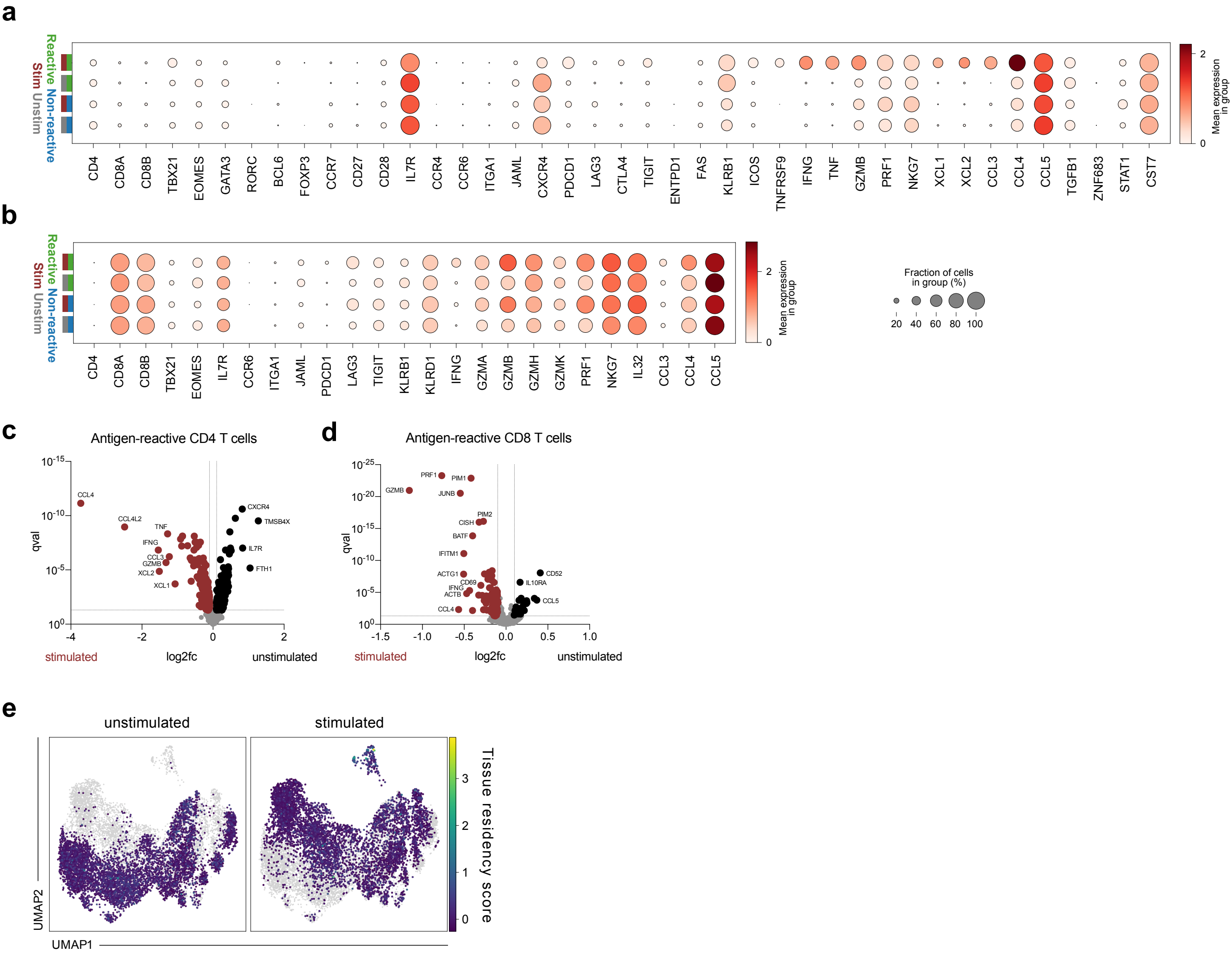

Extended Data Figure 6

Supplementary Figure 6. Ex vivo and stimulation-biased phenotypes of antigen-reactive T cells. a, b, Dot plots of log-normalized expression of selected marker genes by clonotype group (reactive and non-reactive) and by condition (stimulated: 'stim', unstimulated: 'unstim') for CD4 T cells (**a**) (stimulated reactive n=68, unstimulated reactive n=178, unstimulated nonreactive n=539, stimulated nonreactive n=249) and CD8 T cells (**b**) (stimulated reactive n=181, unstimulated reactive n=366, unstimulated nonreactive n=2,711, stimulated nonreactive n=1,305). **c, d,** Volcano plots of differential expression test of stimulated condition versus unstimulated condition in antigen reactive CD4 T cells (**c**) and CD8 T cells (**d**). **e,** UMAPs of all T cells with tissue residency score superimposed. For definition of tissue residency scores see methods section as well as reference[1]. Data are shown for patient GT_3 (n=11,460 cells in total).

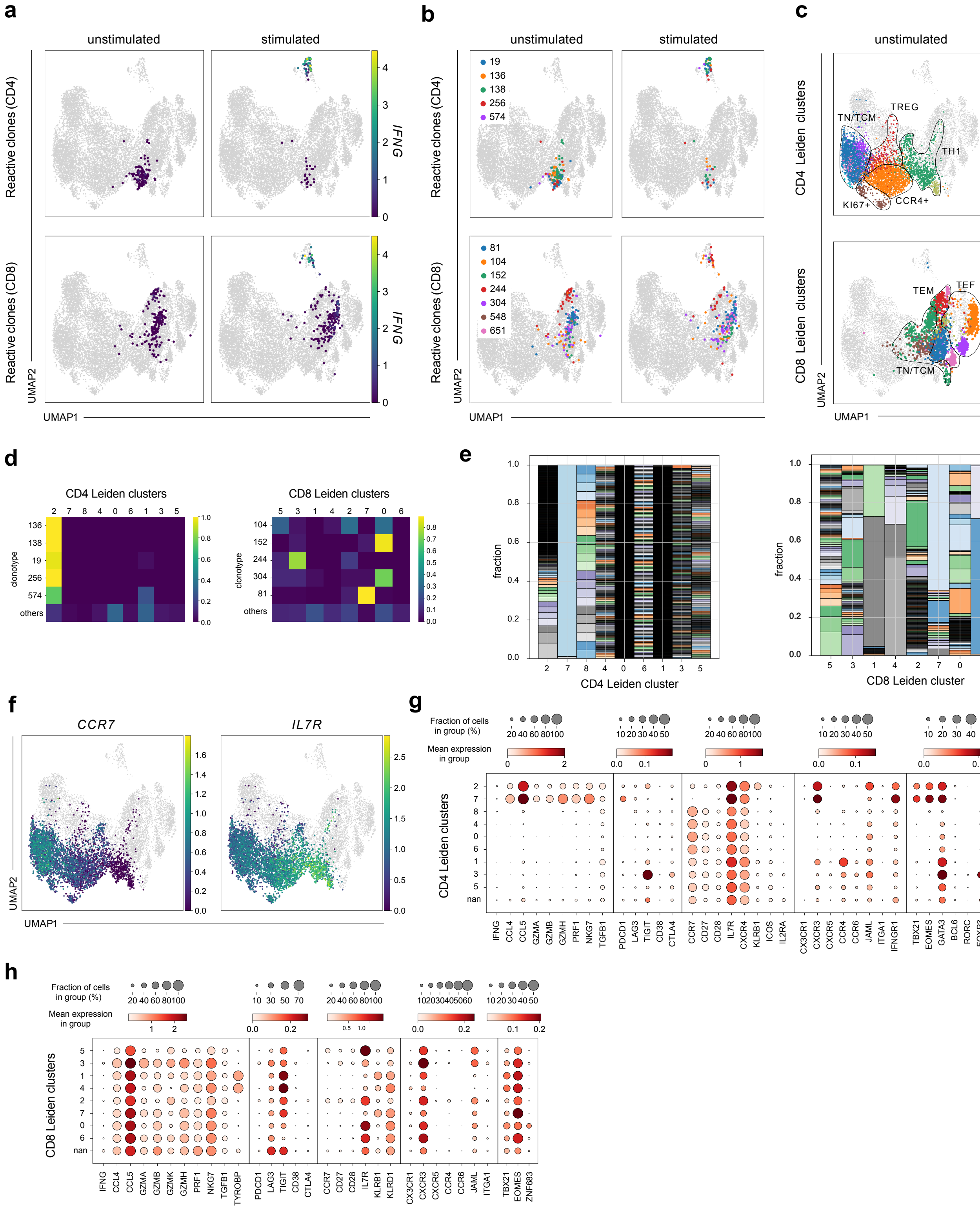

Extended Data Figure 7

Supplementary Figure 7. Phenotypic characterization of unstimulated T cells. a, b, UMAPs of all T cells (n=11,460 cells in total), with cells from reactive clonotypes highlighted by cell type and with log-normalized *IFNG* expression (**a**) or clonotype identity (**b**) superimposed. **c,** UMAPs of all T cells (n=11,460 cells in total) from unstimulated condition highlighted and cell type-specific (CD4 or CD8) Leiden cluster annotation superimposed (UMAPs are identical to some of the UMAPs shown in Fig. 3c, but are shown here again for the sake of clarity (b-c)). **d,** Fraction of cells per clonotype in each Leiden cluster from (c) for both CD4 and CD8 T cells. **e,** Stacked bar plots for each CD4 (left panel) and CD8 (right panel) Leiden clusters, in which each rectangle describes the proportion of the cells of one clonotype out of all cells in that cluster. Box boundaries and all-black stacks indicate very narrow boxes and diverse clonotypes. **f,** UMAPs of all T cells (n=11460 cells in total) with unstimulated CD4 T cells highlighted and log-normalized *CCR7* and *IL7R* expression superimposed. **g, h,** Dot plots of log-normalized expression of selected marker genes by cell type-specific Leiden cluster for CD4 T cells (n=7,558 cells) (**g**) and CD8 T cells (n=3,902 cells) (**h**). Data are shown for patient GT_3.

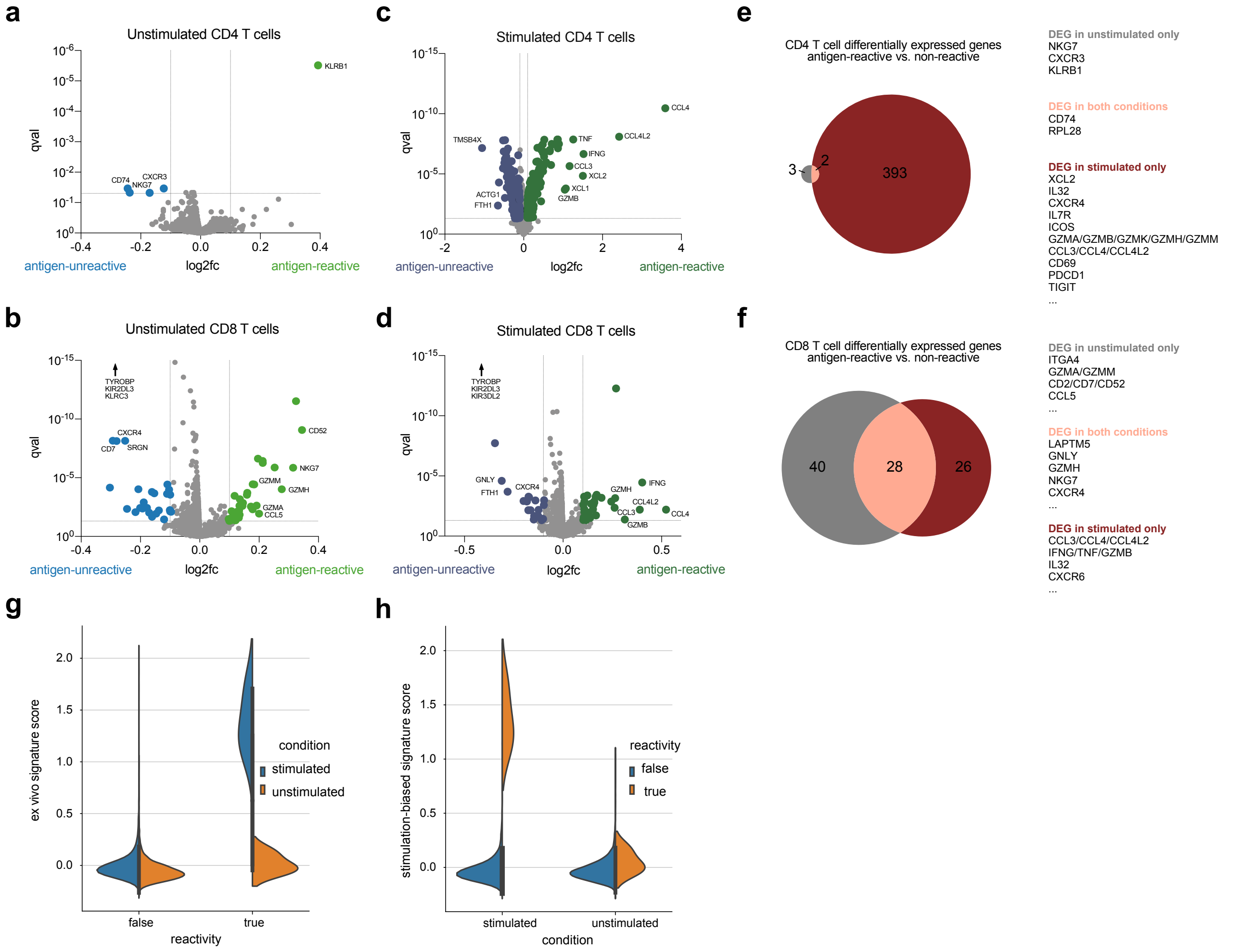

Extended Data Figure 8

Supplementary Figure 8. Ex vivo and stimulation-biased phenotypic signature scores of antigen-reactive T cells. a-d, Volcano plots of differential expression test of cells from non-reactive versus reactive clonotypes in unstimulated CD4 (**a**) and CD8 (**b**) T cells, and stimulated CD4 (**c**) and CD8 (**d**) T cells. For the sake of clarity, *TYROBP* (l2fc -0.48; qval $7x10^{-131}$), *KIR2DL3* (l2fc -0.15; qval $3x10^{-60}$) and *KLRC3* (l2fc -0.13; qval $3x10^{-55}$) are not displayed for unstimulated CD8 T cells, as well as *TYROBP* (l2fc -0.45; qval $1x10^{-110}$), *KIR2DL3* (l2fc -0.25; qval $1x10^{-78}$) and *KIR3DL2* (l2fc -0.10; qval $8x10^{-22}$) for stimulated CD8 T cells. **e, f,** Venn diagrams of overlaps of differentially expressed genes between tests from panel a, c (**e**) and b, d (**f**). Data are shown for patient GT_3. **g, h,** Violin plots of scores applied to GT_2 based on gene sets defined on GT_3. The distributions are shown separately for each stimulation condition and for reactive and non-reactive clonotypes separately. The score shown is the difference between conditions in reactive cells (‘ex vivo signature score’, **g**) and the difference between reactive and unreactive cells in stimulated cells (‘stimulation-biased signature score’, **h**).

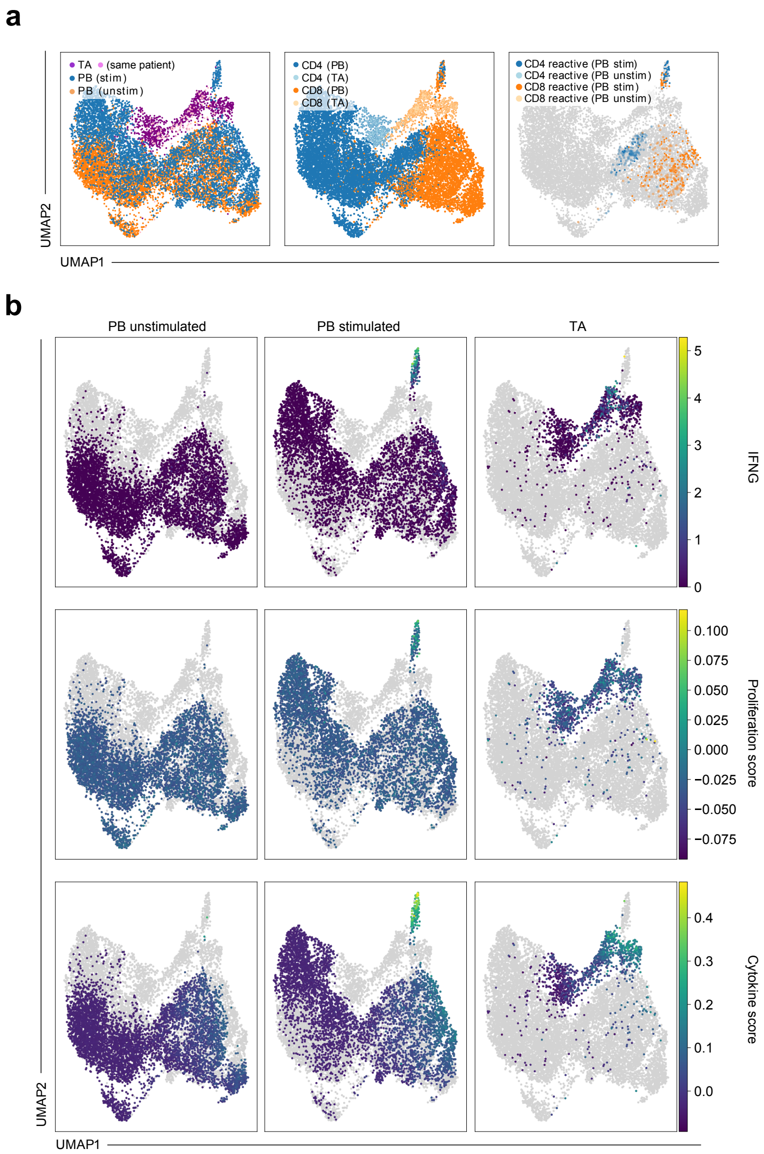

Extended Data Figure 9

Supplementary Figure 9. Characterization of cells from tracheal aspirate samples in context of stimulated and unstimulated PB T cells. a, UMAP of all PB T cells (stimulated and unstimulated, n=12,503 cells) of patient GT_3 and cells from tracheal aspirate samples (TA) with sample, cell type and reactivity label in superimposed. **b,** UMAP as in a) with *IFNG* expression, proliferation score and cytokine score superimposed per data subset.

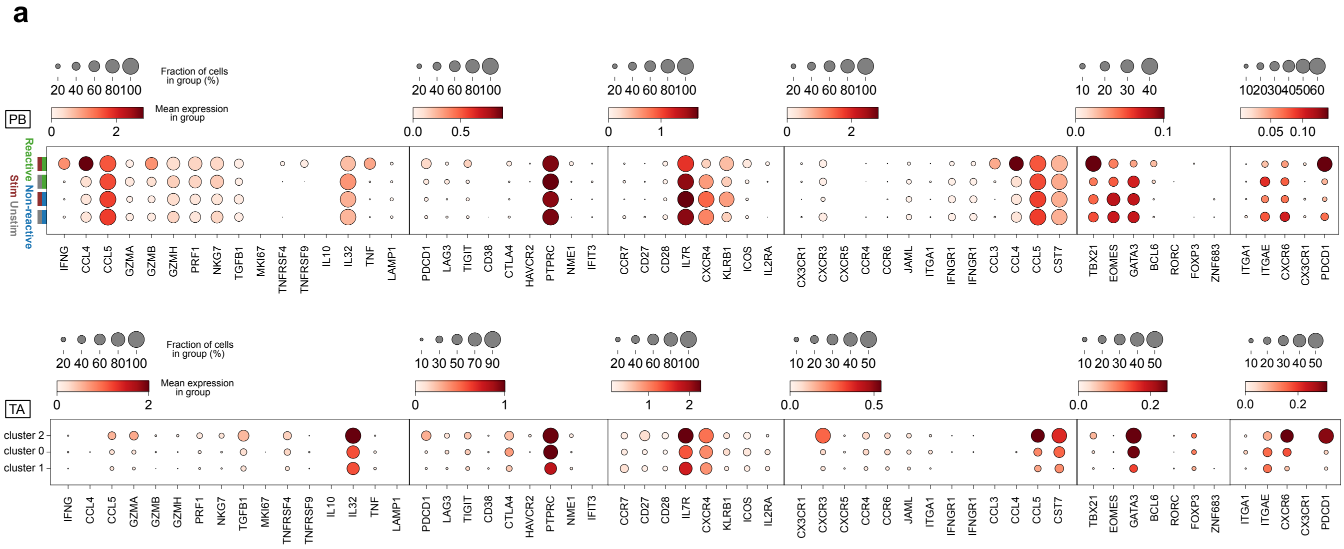

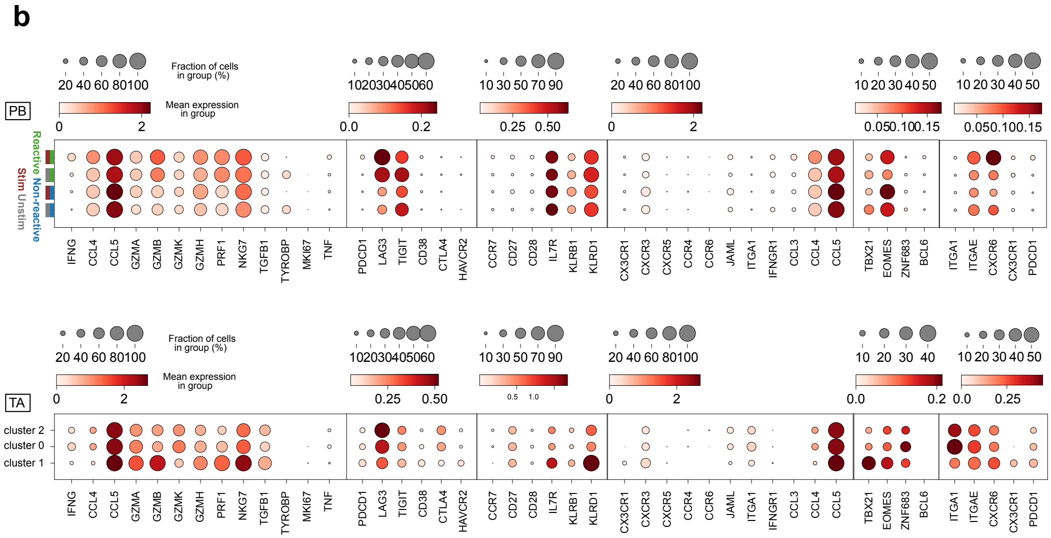

Extended Data Figure 10

Supplementary Figure 10. Characterization of clusters of cells from tracheal aspirate in context of stimulated and unstimulated PB T cells. Shown are dot plots for CD4 T cells (n=530 cells) (**a)** and CD8 T cells (n=696 cells) **(b)**. The upper dot plot in each panel contains grouping of PB T cells into reactive and non-reactive T cells within each condition (stimulated and unstimulated). The lower dot plot in each panel contains a clustering of the tracheal aspirate T cells.

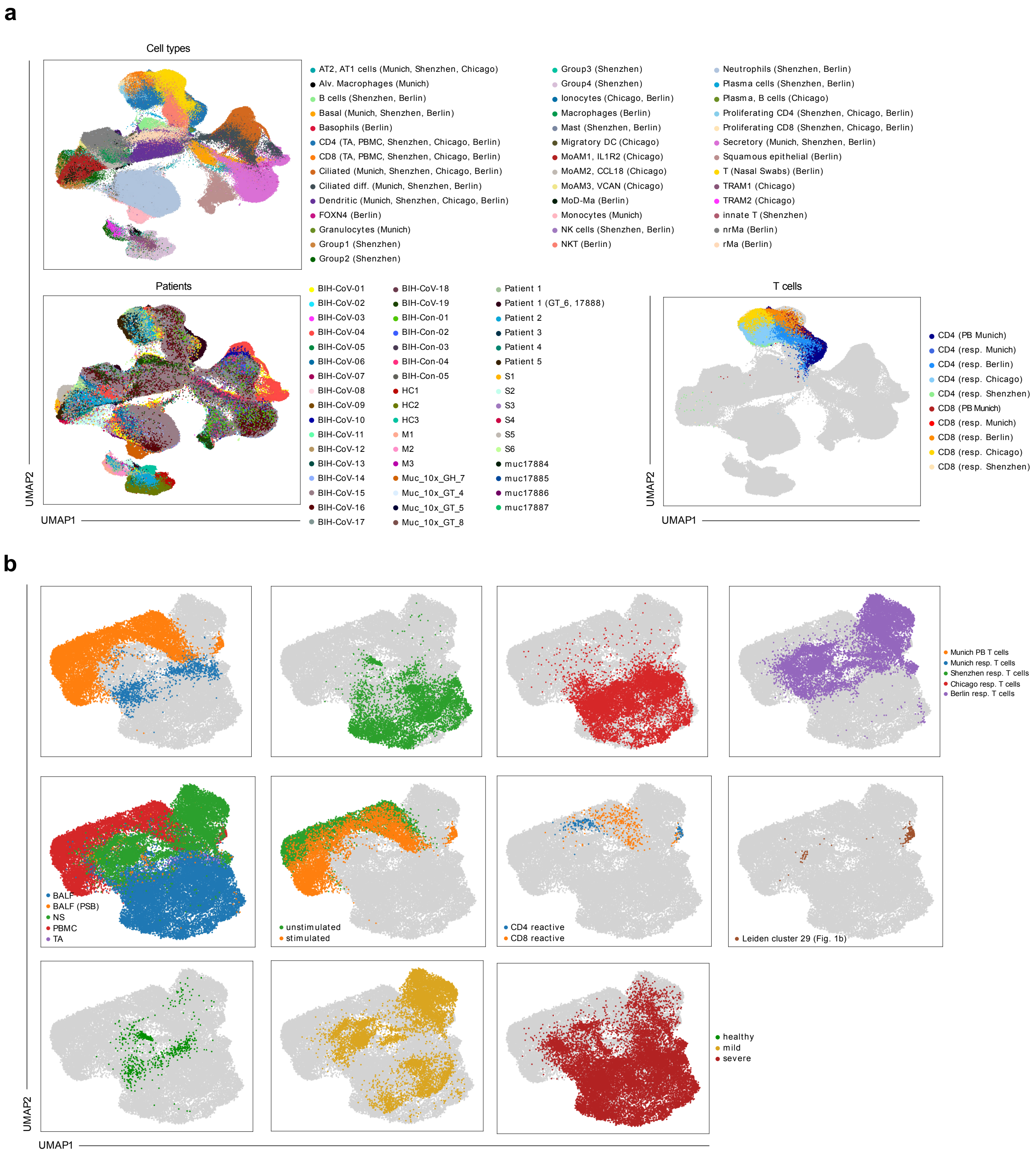

Extended Data Figure 11

Supplementary Figure 11. Joined embedding of cells from all cohorts. a, UMAP computed on all cells from samples from PB and respiratory tract samples of Munich, Chicago, Shenzhen and Berlin cohorts with cell type and sample (top), patient (bottom left) and highlighted T cells (bottom right) superimposed (n=279,663 cells), **b,** UMAP computed on all T cells (n=39,258 cells) from all samples – including nasal swabs – with cohort, sample source, condition, reactivity labels in PB T cells, IFNG+ cluster annotation from PB T cell analysis (Leiden cluster 29) and disease state in other cohorts (healthy, mild, severe) superimposed (from top to bottom and left to right).

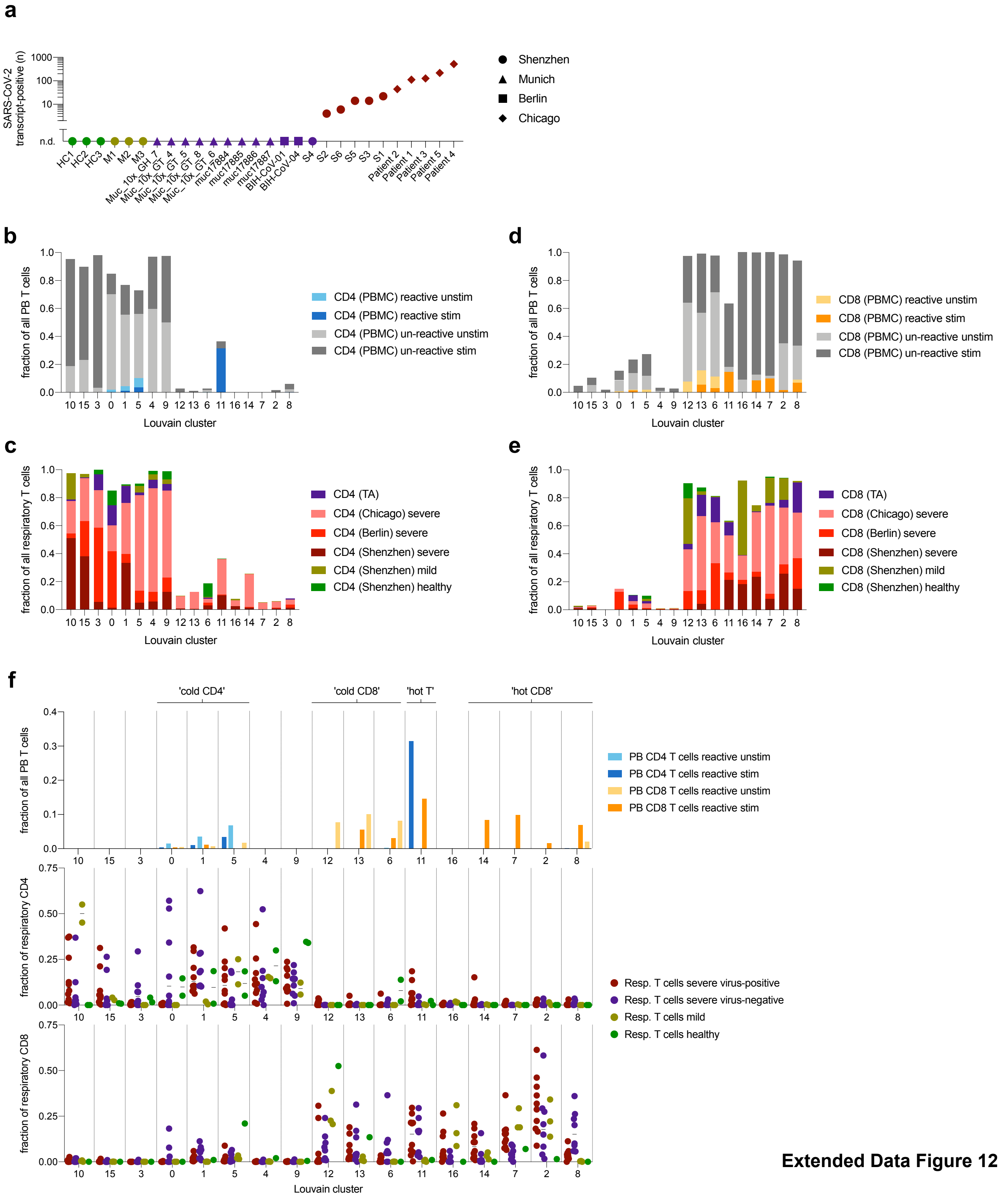

Extended Data Figure 12

Supplementary Figure 12. Characterization of clustering of joined T cell data set. a, Number of SARS-CoV-2 transcript-positive cells per patient among all (including non-T) cells in indicated scRNA seq data. **b-e,** Shown are fractions of cells with particular phenotypes per Louvain clustering, after clustering on all cells from samples from PB and respiratory tract samples of Munich, Chicago, Shenzhen and Berlin cohorts. Shown are reactive and un-reactive cells in stimulated (stim) and unstimulated (unstim) condition for both CD4 (b) and CD8 (c) PB T cells and fraction of cells in cohort for each CD4 (d) and CD8 (e) respiratory tract T cells. **f,** Fractions of all PB (top panel), of respiratory CD4 (middle panel) or of respiratory CD8 (bottom panel) T cells in individual patients (dots) from indicated four disease stages per indicated Louvain cluster. Patients represented by dots; n=1-2 healthy, n=2-3 mild, n=8 severe virus-negative, n=10 severe virus-positive; with less than < 20 cells for each panel were excluded from analysis.

a

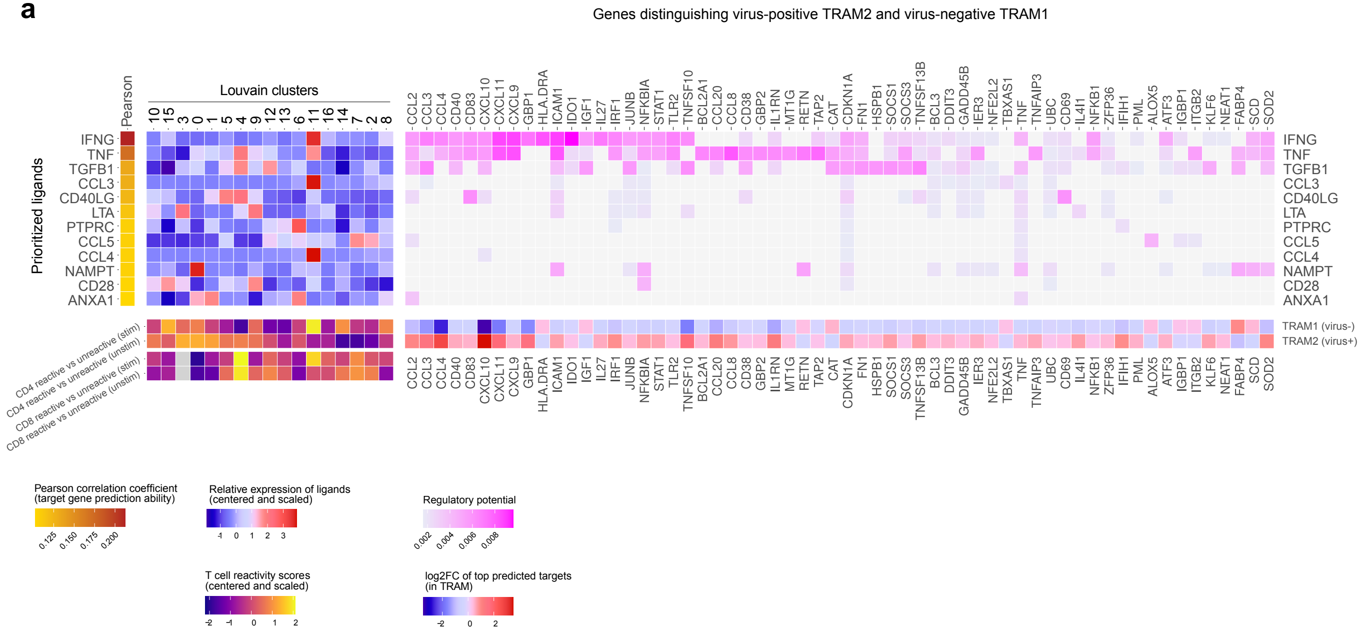

b

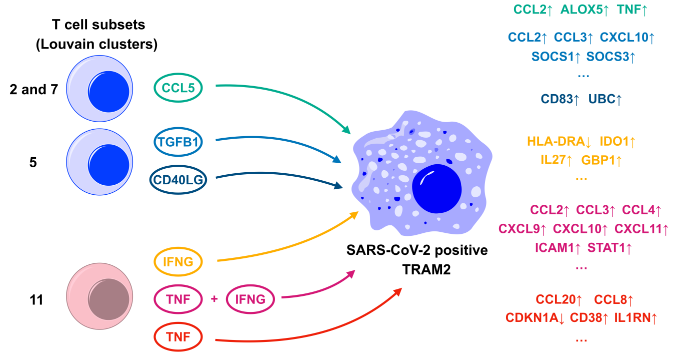

Extended Data Figure 13

Supplementary Figure 13. Cell-cell communication between individual T cell subsets and Tissue-resident Macrophages (TRAMs) from the respiratory tract of patients with severe COVID-19 in the scRNA-seq reference cohort from Chicago. a, Heatmap representation of the NicheNet analysis[2] of ligand-target pairs regulating genes differentially expressed genes between virus-negative TRAM1 and virus-positive TRAM2. From left to right: (1) potential ligands expressed by respiratory tract T cells ranked according to their ability (Pearson correlation coefficient) to predict the gene expression changes observed between TRAM1 and TRAM2, (2) relative expression of the top ranked ligands in individual T cell subsets (Louvain clusters identified by unsupervised clustering of the integrated data set, see Fig. 4b) (top) and mean 'ex vivo signature scores' per individual T cell subset (stimulated: 'stim', unstimulated: 'unstim'), (3) potential targets of the top ranked ligands and their regulatory potential in TRAMs (top) and their log2-fold change in gene expression between TRAM1and TRAM2 (bottom). **b,** Graphical summary of results from (a) with exemplary sender cell groups/ligands and target genes.

a

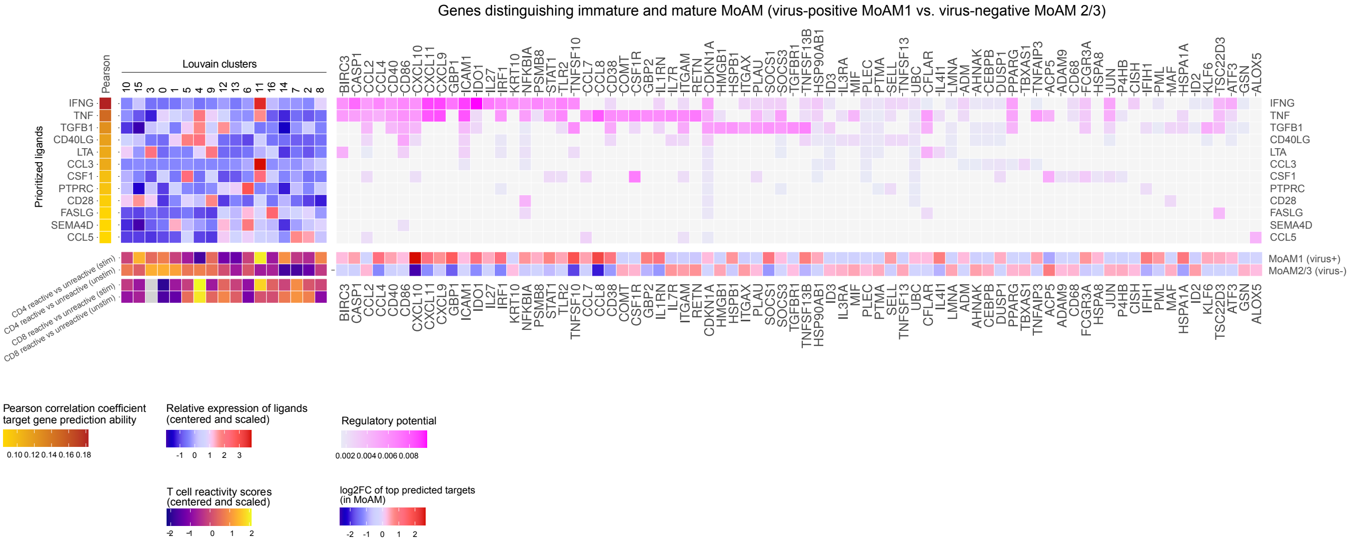

b

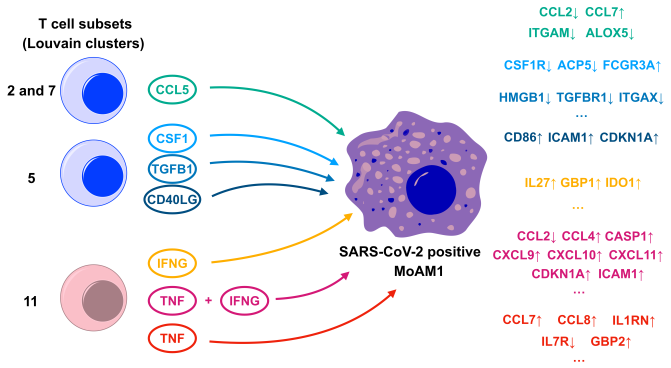

Extended Data Figure 14

Supplementary Figure 14. Cell-cell communication between individual T cell subsets and Monocyte-derived Macrophages (MoAMs) from the respiratory tract of patients with severe COVID-19 in the scRNA seq reference cohort from Chicago. a, Heatmap representation of the NicheNet analysis[2] of ligand-target pairs regulating genes differentially expressed genes between virus-negative MoAM2/3 and virus-positive MoAM1. From left to right: (1) potential ligands expressed by respiratory tract T cells ranked according to their ability (Pearson correlation coefficient) to predict the gene expression changes observed between virus-negative MoAM2/3 and virus-positive MoAM1, (2) relative expression of the top ranked ligands in individual T cell subsets (Louvain clusters identified by unsupervised clustering of the integrated data set, see Fig. 4b) (top) and mean 'ex vivo signature scores' per individual T cell subset (stimulated: 'stim', unstimulated: 'unstim'), (3) potential targets of the top ranked ligands and their regulatory potential in MoAMs (top) and their log2-fold change in gene expression between virus-negative MoAM2/3 and virus-positive MoAM1 (bottom). **b,** Graphical summary of results from (a) with exemplary sender cell groups/ligands and target genes.

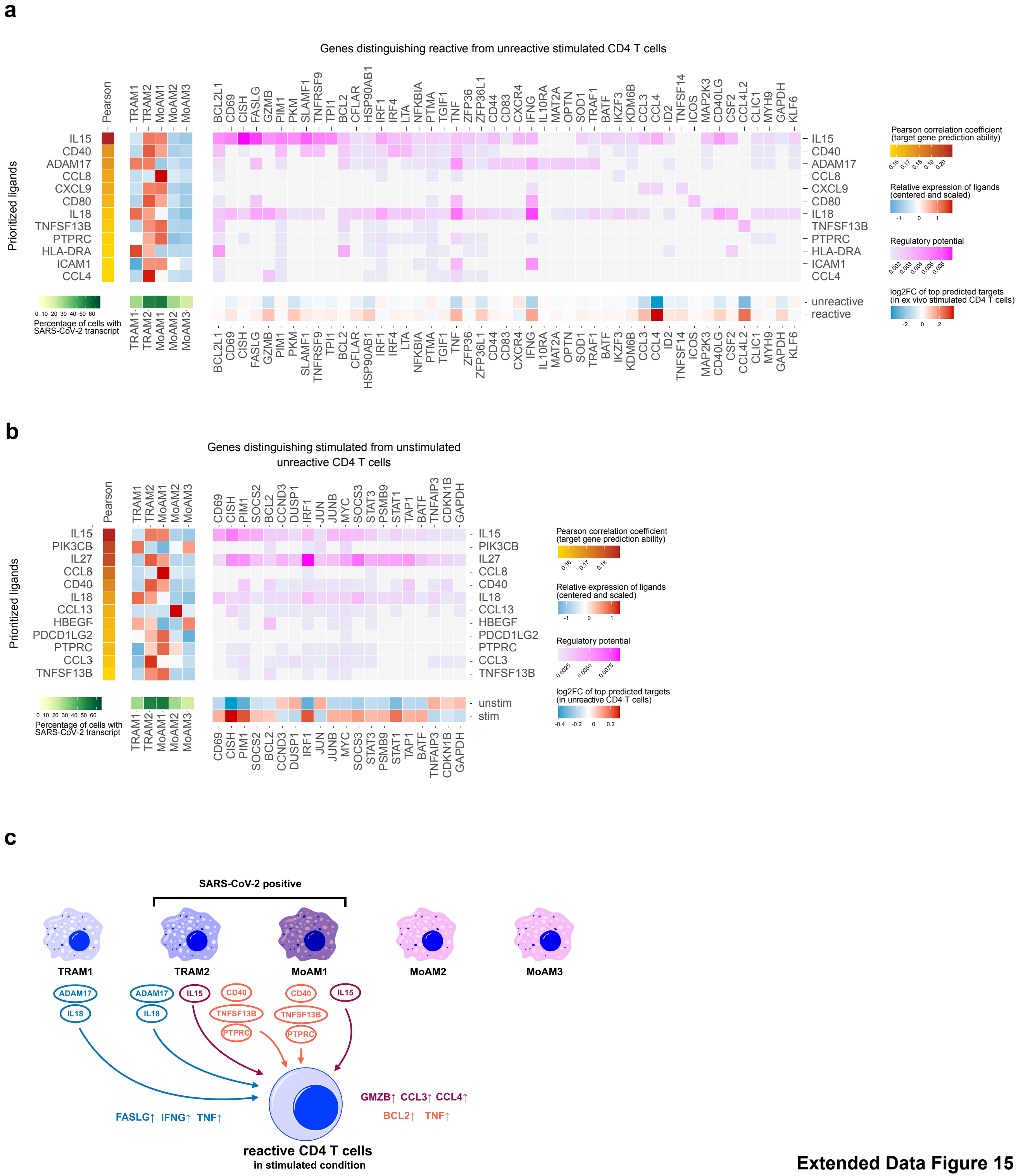

Extended Data Figure 15

Supplementary Figure 15. Cell-cell communication between macrophage subpopulations and CD4 T cells from patients with severe COVID-19 in the scRNA seq reference cohort from Chicago. a, b, Heatmap representation of the NicheNet analysis[2] of ligand-target pairs regulating genes differentially expressed genes between **(a)** reactive and unreactive CD4 T cells after *in vitro* stimulation SARS-CoV-2 spike protein peptide mix and, to obtain an overview on unspecific 'bystander' effects present in an inflammatory environment, **(b)** stimulated and unstimulated unreactive CD 4 T cells. From left to right: (1) potential ligands expressed by macrophages ranked according to their ability (Pearson correlation coefficient) to predict the gene expression changes observed *in vitro*, (2) relative expression of the top ranked ligands in macrophage subpopulations (top) and the percentage of SARS-CoV-2 infected cells (SARS-CoV-2 genes per cell ≥ 1) per macrophage subpopulation (bottom), (3) potential targets of the top ranked ligands and their regulatory potential in CD4 T cells (top) and their log2-fold change in gene expression (bottom) between **(a)** reactive and unreactive *in vitro* stimulated CD4 T cells and **(b)** stimulated and unstimulated unreactive CD4 T cells. **c,** Graphical summary of results from (a) with exemplary ligands and target genes.

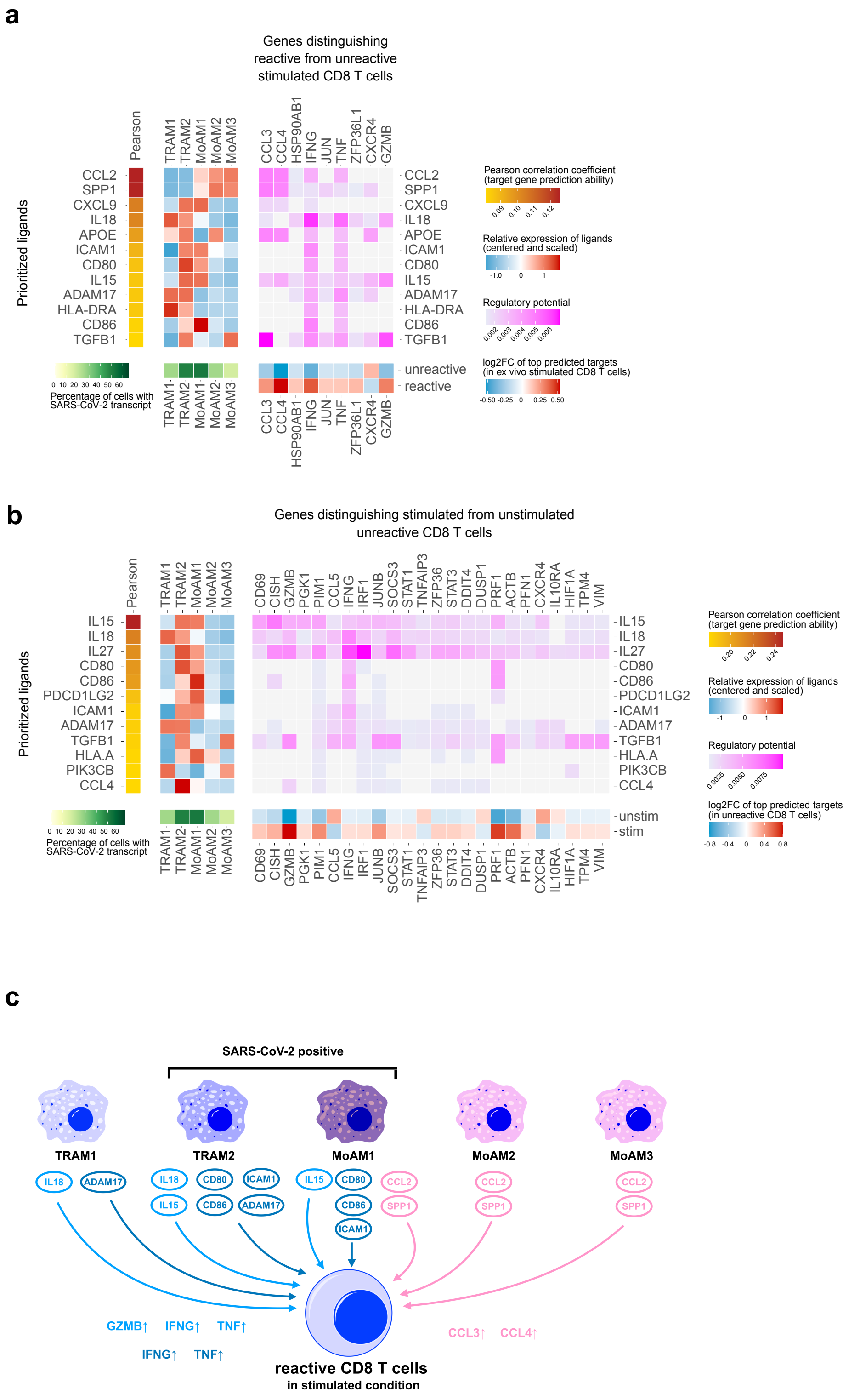

Extended Data Figure 16

Supplementary Figure 16. Cell-cell communication between macrophage subpopulations and CD8 T cells from patients with severe COVID-19 in the scRNA seq reference cohort from Chicago. a, b, Heatmap representation of the NicheNet analysis[2] of ligand-target pairs regulating genes differentially expressed genes between **(a)** reactive and unreactive CD8 T cells after *in vitro* stimulation SARS-CoV-2 spike antigen peptide mix and, to obtain an overview on unspecific 'bystander' effects present in an inflammatory environment, **(b)** stimulated and unstimulated unreactive CD 4 T cells. From left to right: (1) potential ligands expressed by macrophages ranked according to their ability (Pearson correlation coefficient) to predict the gene expression changes observed *in vitro*, (2) relative expression of the top ranked ligands in macrophage subpopulations (top) and the percentage of SARS-CoV-2 infected cells (SARS-CoV-2 genes per cell ≥ 1) per macrophage subpopulation (bottom), (3) potential targets of the top ranked ligands and their regulatory potential in CD8 T cells (top) and their log2-fold change in gene expression (bottom) between **(a)** reactive and unreactive *in vitro* stimulated CD8 T cells and **(b)** stimulated and unstimulated unreactive CD8 T cells. **c,** Graphical summary of results from (a) with exemplary ligands and target genes.

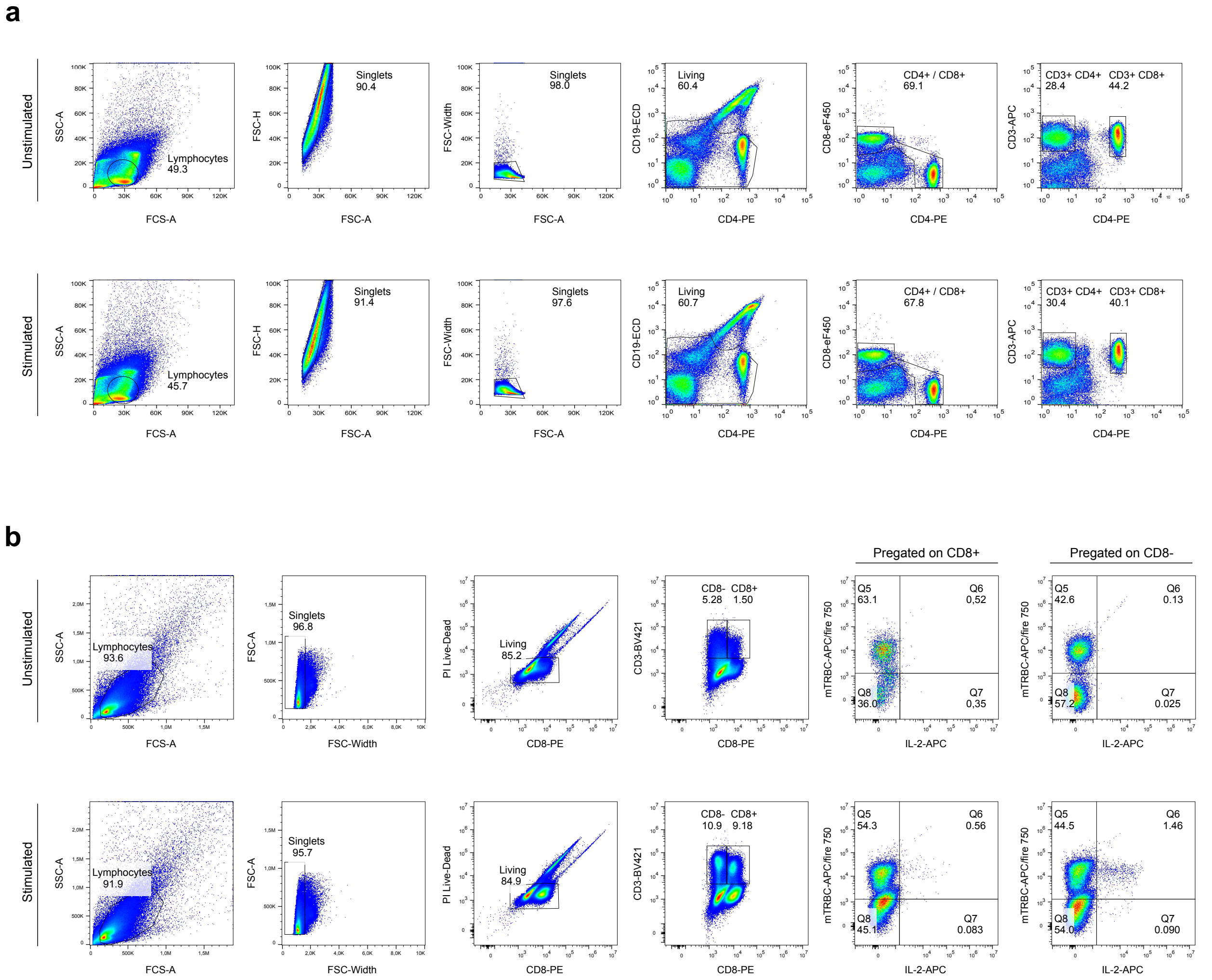

Extended Data Figure 17

Supplementary Figure 17. Gating strategy. a, Representative gating strategy for flow cytometry-assisted cell sorting prior to scRNA seq. **b,** Representative gating strategy for flow cytometric analysis of T cells engineered with TCRs predicted to be reactive. 'Stimulated' indicates stimulation with SARS-CoV-2 spike antigen. Hierarchical gating was performed from left to right.

Supplementary References

1. Kumar, B. V. *et al.* Human Tissue-Resident Memory T Cells Are Defined by Core Transcriptional and Functional Signatures in Lymphoid and Mucosal Sites. *Cell Rep.* **20**, 2921–2934 (2017).
2. Browaeys, R., Saelens, W. & Saeys, Y. NicheNet: modeling intercellular communication by linking ligands to target genes. *Nat. Methods* **17**, 159–162 (2020).
