# Supplementary material for: Single-cell RNA sequencing reveals ex vivo signatures of SARS-CoV-2-reactive T cells through ‘reverse phenotyping’
Source: Nat Commun. 2021 Jul 26;12:4515. doi: 10.1038/s41467-021-24730-4 (PMC8313584; doi:10.1038/s41467-021-24730-4)
Supplement: Supplementary file 2 — Description of Additional Supplementary Files [file 41467_2021_24730_MOESM2_ESM.pdf]

Description of Additional Supplementary Files

Supplementary Data 1: Clinical information on patients from Munich cohort

Supplementary Data 2: Differential gene expression results of CD8 T cells in IFNG cluster compared with other CD8 T cells in stimulated condition

Supplementary Data 3: Differential gene expression results of CD4 T cells in IFNG cluster compared with other CD4 T cells in stimulated condition

Supplementary Data 4: Differential gene expression results of SARS-CoV-2-reactive CD4 T cells unstimulated versus stimulated condition

Supplementary Data 5: Differential gene expression results of SARS-CoV-2-reactive CD8 T cells unstimulated versus stimulated condition

Supplementary Data 6: Differential gene expression results of unstimulated CD4 T cells between SARS-CoV-2-reactive and non-reactive cells

Supplementary Data 7: Differential gene expression results of unstimulated CD8 T cells between SARS-CoV-2-reactive and non-reactive cells

Supplementary Data 8: Differential gene expression results of stimulated CD4 T cells between SARS-CoV-2-reactive and non-reactive cells

Supplementary Data 9: Differential gene expression results of stimulated CD8 T cells between SARS-CoV-2-reactive and non-reactive cells

Supplementary Data 10: Clonotypes selected for orthotopic TCR replacement

Supplementary Data 11: Clonotypes discovered in GT_2 after filtering

Supplementary Data 12: Clonotypes discovered in GT_3 after filtering

Supplementary Data 13: Sequences of primers to amplify TCR targeting constructs for OTR
