# Supplementary material for: Single-cell RNA sequencing reveals ex vivo signatures of SARS-CoV-2-reactive T cells through ‘reverse phenotyping’
Source: Nat Commun. 2021 Jul 26;12:4515. doi: 10.1038/s41467-021-24730-4 (PMC8313584; doi:10.1038/s41467-021-24730-4)
Supplement: Supplementary file 16 — Reporting summary [file 41467_2021_24730_MOESM16_ESM.pdf]

nature research

Corresponding author(s): Herbert B. Schiller & Kilian Schober

Last updated by author(s): May 28, 2021

Reporting Summary

Nature Research wishes to improve the reproducibility of the work that we publish. This form provides structure for consistency and transparency in reporting. For further information on Nature Research policies, see our Editorial Policies and the Editorial Policy Checklist.

Statistics

For all statistical analyses, confirm that the following items are present in the figure legend, table legend, main text, or Methods section.

n/a	Confirmed	
☐	☒	The exact sample size (n) for each experimental group/condition, given as a discrete number and unit of measurement
☐	☒	A statement on whether measurements were taken from distinct samples or whether the same sample was measured repeatedly
☐	☒	The statistical test(s) used AND whether they are one- or two-sided<br>*Only common tests should be described solely by name; describe more complex techniques in the Methods section.*
☐	☒	A description of all covariates tested
☐	☒	A description of any assumptions or corrections, such as tests of normality and adjustment for multiple comparisons
☐	☒	A full description of the statistical parameters including central tendency (e.g. means) or other basic estimates (e.g. regression coefficient) AND variation (e.g. standard deviation) or associated estimates of uncertainty (e.g. confidence intervals)
☐	☒	For null hypothesis testing, the test statistic (e.g. F, t, r) with confidence intervals, effect sizes, degrees of freedom and P value noted<br>*Give P values as exact values whenever suitable.*
☒	☐	For Bayesian analysis, information on the choice of priors and Markov chain Monte Carlo settings
☐	☒	For hierarchical and complex designs, identification of the appropriate level for tests and full reporting of outcomes
☐	☒	Estimates of effect sizes (e.g. Cohen's d, Pearson's r), indicating how they were calculated

Our web collection on statistics for biologists contains articles on many of the points above.

Software and code

Policy information about availability of computer code

Data collection	No software was used for data collection.
Data analysis	Flow-cytometric data were analyzed with FlowJo v10.4.2. For visualization and some statistical analysis, GraphPadPrism (v8.4.3) was used. We performed analysis of scRNA seq data with CellRanger version 3.1.0 (10X Genomics), scanpy (v1.6.0), scirpy (v0.4), diffxpy (v.0.7.4), SoupX (v1.3.6). Some hierarchical clustering analyes were performed with Morpheus (https://software.broadinstitute.org/morpheus; no version number indicated; version used as of September 2020). NicheNet analyses were performed with R (version 3.6.3) packages nichenetr (version 1.0) and Seurat (version 3.2). For code, see Extended Data File 1.

For manuscripts utilizing custom algorithms or software that are central to the research but not yet described in published literature, software must be made available to editors and reviewers. We strongly encourage code deposition in a community repository (e.g. GitHub). See the Nature Research guidelines for submitting code & software for further information.

Data

Policy information about availability of data

All manuscripts must include a data availability statement. This statement should provide the following information, where applicable:

- Accession codes, unique identifiers, or web links for publicly available datasets
- A list of figures that have associated raw data
- A description of any restrictions on data availability

All data generated or analyzed during this study are included in this article, its supplementary information files and are available at NCBI GEO under the accession number GSE171037 (https://www.ncbi.nlm.nih.gov/geo/query/acc.cgi?acc=GSE171037). Additional raw data are available from the corresponding authors upon reasonable request.

The three independent reference cohorts were accessed as follows:
“Shenzhen”: Single-cell landscape of bronchoalveolar immune cells in patients with COVID-19 [Liao et al.48]: All data used in this study, including scRNA-seq and scTCR-seq raw data, filtered expression matrix and scTCR-seq contig annotation that support the findings of this study can be accessed in GEO under the accession number GSE145926.
https://www.ncbi.nlm.nih.gov/geo/query/acc.cgi?acc=GSE145926

“Chicago”: Alveolitis in severe SARS-CoV-2 pneumonia is driven by self-sustaining circuits between infected alveolar macrophages and T cells [Grant et al.49]
Single-cell RNA-seq: Counts tables and integrated objects are available through GEO with accession number GSE155249.
https://www.ncbi.nlm.nih.gov/geo/query/acc.cgi?acc=GSE155249

“Berlin”: COVID-19 severity correlates with airway epithelium-immune cell interactions identified by single-cell analysis [Chua et al.47]
In addition, count and metadata tables containing patient identification, sex, age, cell type and quality control metrics for each cell are available at FigShare: https://doi.org/10.6084/m9.figshare.12436517.
https://figshare.com/articles/COVID-19_severity_correlates_with_airway_epithelium-immune_cell_interactions_identified_by_single-cell_analysis/12436517

Field-specific reporting

Please select the one below that is the best fit for your research. If you are not sure, read the appropriate sections before making your selection.

- [x] Life sciences
- [ ] Behavioural & social sciences
- [ ] Ecological, evolutionary & environmental sciences

For a reference copy of the document with all sections, see nature.com/documents/nr-reporting-summary-flat.pdf

Life sciences study design

All studies must disclose on these points even when the disclosure is negative.

Sample size	Sample sizes were chosen on the basis of preliminary experiments. No power analysis was performed before experiments. ScRNA seq per condition and donor was performed with 10.000 cells. We initially suspected that antigen-reactive clones would make up at least 0.1% of all T cells, which would be equivalent to 10 cells in this setting.
Data exclusions	In general, no data were excluded. As mentioned in the respective figure legends, in Fig. 4c and Ext. Data Fig. 12f, patients with less than 20 cells in Louvain cluster 11 were excluded from analysis since, in these patients, each single cell would be equivalent to more than 5% and therefore disproportionately generates outliers.
Replication	ScRNA seq results were confirmed in two different patients. Reactive TCRs were experimentally validated as described. For more information see figure legends.
Randomization	No randomization was performed. Because of the complexity and costs of the experimental approach, PBMC stimulation was performed for two different donors. We suspected that this should be sufficient to provide proof-of-concept for the methodological approach to use stimulation-induced transcriptomic shifts for identification of antigen-reactive T cells and reverse phenotyping.
Blinding	No blinding was performed since the purpose of the study was not a comparison between different donors, but to provide methodological proof-of-concept in both donors.

Reporting for specific materials, systems and methods

We require information from authors about some types of materials, experimental systems and methods used in many studies. Here, indicate whether each material, system or method listed is relevant to your study. If you are not sure if a list item applies to your research, read the appropriate section before selecting a response.

Materials & experimental systems

n/a	Involved in the study	
[ ]	[x]	Antibodies
[x]	[ ]	Eukaryotic cell lines
[x]	[ ]	Palaeontology and archaeology
[x]	[ ]	Animals and other organisms
[ ]	[x]	Human research participants
[x]	[ ]	Clinical data
[x]	[ ]	Dual use research of concern

Methods

n/a	Involved in the study	
[x]	[ ]	ChIP-seq
[ ]	[x]	Flow cytometry
[x]	[ ]	MRI-based neuroimaging

Antibodies

Antibodies used	Experiment (Specificity Target Fluorochrome Company Identifier Dilution)

Sort
human CD8 eF450 Life Technologies 48-0086-42 1:200
human CD3 APC Life Technologies 17-0038-42 1:200
human CD19 ECD Beckman Coulter A07770 1:100
human CD4 PE Life Technologies MHCD0404 1:100
human CD56 FITC Life Technologies 11-0566-42 1:100
OTR ICCS
human CD3 BV421 BD Bioscience 563797 1:100
human CD8 PE Life Technologies 12-0086-42 1:200
mouse mTRBC APC/fire 750 BioLegend 109246 1:50
human IL-2 APC BD Bioscience 341116 1:25

Validation

Antibody validations were performed by antibody suppliers per quality assurance literature provided by each supplier:
- https://www.thermofisher.com/antibody/product/CD8a-Antibody-clone-OKT8-OKT-8-Monoclonal/48-0086-42
- https://www.thermofisher.com/antibody/product/CD3-Antibody-clone-UCHT1-Monoclonal/17-0038-42
- https://www.beckman.de/reagents/coulter-flow-cytometry/antibodies-and-kits/single-color-antibodies/cd19/a07770
- https://www.thermofisher.com/antibody/product/CD4-Antibody-clone-S3-5-Monoclonal/MHCD0404
- https://www.thermofisher.com/antibody/product/CD56-NCAM-Antibody-clone-TULY56-Monoclonal/11-0566-42
- https://www.bdbiosciences.com/us/applications/research/t-cell-immunology/th-1-cells/surface-markers/human/bv421-mouse-anti-human-cd3-sk7-also-known-as-leu-4/p/563797
- https://www.thermofisher.com/antibody/product/CD8a-Antibody-clone-OKT8-OKT-8-Monoclonal/12-0086-42
- https://www.biolegend.com/en-us/search-results/apc-fire-750-anti-mouse-tcr-beta-chain-antibody-13672
- https://www.bdbiosciences.com/us/applications/research/t-cell-immunology/th-1-cells/intracellular-markers/cytokines-and-chemokines/human/apc-mouse-anti-human-il-2-5344111/p/341116

Human research participants

Policy information about studies involving human research participants

Population characteristics

All Munich cohort patients were PCR-confirmed SARS-CoV-2 positive, admitted to the intensive care unit in the University Hospital of the Ludwig-Maximillians University, Munich (n=5), or the Asklepios Lung Clinic Munich-Gauting, Gauting (n=4), for treatment of severe COVID-19 requiring invasive, mechanical ventilation. For further clinical information see Extended Data Table 1. PBMCs and tracheal aspirate samples were taken at the end of April, 2020.

Recruitment

Ventilated patients with severe COVID-19 were sequentially recruited in the indicated hospitals at the end of April, 2020. Any potential selection bias of the patients should not have any impact on the methodological proof-of-concept provided in this study.

Ethics oversight

Written informed consent was obtained from the donors or their caregivers, usage of the blood samples was approved according to national law by the local Institutional Review Board (Ethikkommission der Medizinischen Fakultät der Ludwigs-Maximilian-Universität München; vote IDs 19-629, 19-630 and 20-259) and/or samples were used according to legal provisions defined by the German Infection Protection Act (IfSG).

Note that full information on the approval of the study protocol must also be provided in the manuscript.

Flow Cytometry

Plots

Confirm that:

- [x] The axis labels state the marker and fluorochrome used (e.g. CD4-FITC).
- [x] The axis scales are clearly visible. Include numbers along axes only for bottom left plot of group (a 'group' is an analysis of identical markers).
- [x] All plots are contour plots with outliers or pseudocolor plots.
- [x] A numerical value for number of cells or percentage (with statistics) is provided.

Methodology

Sample preparation

Peripheral blood mononuclear cells (PBMC) were isolated from EDTA whole blood by gradient density centrifugation according to manufacturer's instructions (Biocoll, Biochrom) and frozen in FCS + 10% DMSO (Merck) for liquid nitrogen storage. After freezing-thawing procedure, T cells were cultured in RPMI 1640 (Gibco) supplemented with 5% human serum, 0.025% l-glutamine, 0.1% HEPES, 0.001% gentamycin and 0.002% streptomycin (hereafter RPMI-HS).
Tracheal aspirates were digested with 4ml dispase (50 units/ml) (Corning, #354235) and 25 µl DNase (30 µg/ml) (Qiagen, #79254) at 37°C for 10 min with occasional shaking. The digestion was then stopped with 10 ml of ice-cold 10% FCS/PBS. To obtain single cell suspensions, the digestion mix was passed through a 70 µm cell strainer. Red blood cell lysis was performed only when necessary by incubating the cells with 3 ml RBL buffer at RT for 1 min.

Instrument

Flow sorting of CD4+ and CD8+ cells from the stimulated and unstimulated condition was conducted on a MoFlo Astrios EQ (Beckman Coulter) under biosafety level 3.
Flow cytometric analyses were acquired on a Cytoflex (S) flow cytometer (Beckman Coulter).

	ScRNA seq library preparation was performed on a 10x Genomics Chromium Controller.<br>Quality control of scRNA seq libraries was performed on a Bioanalyzer 2100.<br>Illumina paired end sequencing was performed with 150 or 200 (3' gene expression) and 100 cycles (5' gene expression and TCR libraries) on a NovaSeq 6000.
Software	For flow cytometry analysis, FlowJo software (FlowJo LLC v10.4.2) was used. For visualization and some statistical analysis, GraphPad Prism (v8.4.3)
Cell population abundance	For each experiment, at least one purity control was performed by re-analysis of sorted cells. Purity was consistently > 98 %.
Gating strategy	Lymphocyte gates were defined according to FSC and SSC. Singlets were gated with FSC and FSC pulse width. Living cells were gated with viability dyes as being not double positive in PE and ECD channels. Additional gating was performed for individual experiments. Gates were set according to mock controls.

☒ Tick this box to confirm that a figure exemplifying the gating strategy is provided in the Supplementary Information.
